# Forest Fire Smoke Exposures and Out-of-Hospital Cardiac Arrests in Melbourne, Australia: A Case-Crossover Study

**DOI:** 10.1289/ehp.1408436

**Published:** 2015-03-20

**Authors:** Martine Dennekamp, Lahn D. Straney, Bircan Erbas, Michael J. Abramson, Melita Keywood, Karen Smith, Malcolm R. Sim, Deborah C. Glass, Anthony Del Monaco, Anjali Haikerwal, Andrew M. Tonkin

**Affiliations:** 1 Department of Epidemiology and Preventive Medicine, Monash University, Melbourne, Victoria, Australia; 2 Department of Public Health, La Trobe University, Melbourne, Victoria, Australia; 3 Centre for Australian Weather and Climate Research, CSIRO (Commonwealth Scientific and Industrial Research Organisation) Marine and Atmospheric Research, Aspendale, Victoria, Australia; 4 Research and Evaluation Department, Ambulance Victoria, Melbourne, Victoria, Australia; 5 Emergency Medicine, University of Western Australia, Perth, Western Australia

## Abstract

**Background:**

Millions of people can potentially be exposed to smoke from forest fires, making this an important public health problem in many countries.

**Objective:**

In this study we aimed to measure the association between out-of-hospital cardiac arrest (OHCA) and forest fire smoke exposures in a large city during a severe forest fire season, and estimate the number of excess OHCAs due to the fire smoke.

**Methods:**

We investigated the association between particulate matter (PM) and other air pollutants and OHCA using a case-crossover study of adults (≥ 35 years of age) in Melbourne, Australia. Conditional logistic regression models were used to derive estimates of the percent change in the rate of OHCA associated with an interquartile range (IQR) increase in exposure. From July 2006 through June 2007, OHCA data were collected from the Victorian Ambulance Cardiac Arrest Registry. Hourly air pollution concentrations and meteorological data were obtained from a central monitoring site.

**Results:**

There were 2,046 OHCAs with presumed cardiac etiology during our study period. Among men during the fire season, greater increases in OHCA were observed with IQR increases in the 48-hr lagged PM with diameter ≤ 2.5 μm (PM_2.5_) (8.05%; 95% CI: 2.30, 14.13%; IQR = 6.1 μg/m^3^) or ≤ 10 μm (PM_10_) (11.1%; 95% CI: 1.55, 21.48%; IQR = 13.7 μg/m^3^) and carbon monoxide (35.7%; 95% CI: 8.98, 68.92%; IQR = 0.3 ppm). There was no significant association between the rate of OHCA and air pollutants among women. One hundred seventy-four “fire-hours” (i.e., hours in which Melbourne’s air quality was affected by forest fire smoke) were identified during 12 days of the 2006/2007 fire season, and 23.9 (95% CI: 3.1, 40.2) excess OHCAs were estimated to occur due to elevations in PM_2.5_ during these fire-hours.

**Conclusions:**

This study found an association between exposure to forest fire smoke and an increase in the rate of OHCA. These findings have implications for public health messages to raise community awareness and for planning of emergency services during forest fire seasons.

**Citation:**

Dennekamp M, Straney LD, Erbas B, Abramson MJ, Keywood M, Smith K, Sim MR, Glass DC, Del Monaco A, Haikerwal A, Tonkin AM. 2015. Forest fire smoke exposures and out-of-hospital cardiac arrests in Melbourne, Australia: a case-crossover study. Environ Health Perspect 123:959–964; http://dx.doi.org/10.1289/ehp.1408436

## Introduction

Millions of people worldwide can potentially be exposed to seasonal high levels of smoke from forest (bush or wild) fires, making this an important public health problem. Because forest fires are predicted to increase in frequency and severity (Confalonieri et al. 2007) and smoke from these fires can travel long distances, it is important to understand the impact of these seasonal high peak smoke concentrations.

The smoke from forest fires consists of many different constituents, but the pollutant most significantly increased during smoke episodes is PM_2.5_ (particles with an aerodynamic diameter ≤ 2.5 μm) (Reisen et al. 2011). On days without forest fire smoke, PM_2.5_ makes up approximately 40% of PM_10_ (particles with an aerodynamic diameter ≤ 10 μm) (Chan et al. 2008). During forest fires, this proportion increases dramatically (Reisen et al. 2013). Studies around the world have observed particulate matter (PM) concentrations during forest fires well above the recommended air quality standards (Johnston et al. 2011; Morgan et al. 2010; Reisen et al. 2011, 2013; Sapkota et al. 2005; Schranz et al. 2010). The World Health Organization (2006) 24-hr average air quality guideline for PM_10_ is 50 μg/m^3^ and for PM_2.5_ 25 μg/m^3^. Other pollutants that are increased during forest fire smoke episodes, but not to the extent of PM_2.5_, are ozone (O_3_) and carbon monoxide (CO) (Dutkiewicz et al. 2011; Reisen et al. 2011).

A recent review concluded that several studies have found associations between forest fire smoke and respiratory morbidity (Dennekamp and Abramson 2011); however, only a few studies have investigated cardiovascular health outcomes. Of those studies investigating cardiovascular outcomes and forest fire smoke, most of them investigated hospital admissions (Delfino et al. 2009; Hanigan et al. 2008; Henderson et al. 2011; Johnston et al. 2007; Martin et al. 2013; Morgan et al. 2010; Mott et al. 2005). These studies either showed no association or inconsistent results. Two of the studies have found a weak association with hospital admissions, but only for indigenous people (Hanigan et al. 2008; Johnston et al. 2007). A comprehensive study from Sydney, Australia, found a small increase in nonaccidental mortality at a lag of 1 day after exposure to forest fire smoke [odds ratio (OR) = 1.05; 95% confidence interval (CI): 1.00, 1.10] (Johnston et al. 2011).

We have previously shown an association between urban PM_2.5_ concentrations and out-of-hospital cardiac arrest (OHCA) (Dennekamp et al. 2010; Straney et al. 2014). This poses the question: What are the health effects of exposure to episodes of forest fire smoke, where the PM_2.5_ concentrations may be many times higher than urban background concentrations and when air quality standards are regularly exceeded?

To our knowledge, this is the first study to investigate the association between OHCA and forest fire smoke exposure. OHCA is potentially a better outcome to investigate than hospital admissions, because most patients who present with a cardiac arrest with presumed cardiac etiology die before reaching a hospital (Stub et al. 2011). As a result, it is likely that hospital studies will miss a substantial number of relevant cases of acute cardiac events.

The southeast of Australia experienced a very severe forest fire season in the summer of 2006–2007, and > 1 million hectares of land were burnt. Smoke from the fires traveled long distances and covered the city of Melbourne on several days. This, together with a detailed ambulance registry on OHCAs in Melbourne, provided a unique opportunity to investigate the association between forest fire smoke and cardiac arrests in a large urban population during a severe forest fire season.

## Methods

*Study population and outcome data*. OHCA data covering July 2006 through June 2007 were drawn from Ambulance Victoria’s Victorian Ambulance Cardiac Arrest Registry (VACAR) (http://www.ambulance.vic.gov.au/Research/Latest-Research.html). Ambulance Victoria follows the Utstein style criteria, an established set of common definitions for cardiac arrest (Cummins et al. 1991; Jacobs et al. 2004). The VACAR captures all cardiac arrests attended by the ambulance service (Fridman et al. 2007); it is one of the largest and most comprehensive cardiac registries in the world and includes data on age, sex, and exact time of the emergency call on an individual basis.

OHCAs were included if they occurred in metropolitan Melbourne, had presumed cardiac etiology, and occurred in those ≥ 35 years of age. We excluded those < 35 years of age because it was more difficult to determine possible cardiac etiology (e.g., due to genetic diseases) (Deasy et al. 2011). Of the total number of OHCAs attended by ambulance personnel, about 80% had presumed cardiac etiology and were included in the analysis (Dennekamp et al. 2010). Reasons for exclusions have been detailed elsewhere (Dennekamp et al. 2010), but included OHCAs due to road traffic accidents or other trauma, overdoses, terminal illness, or an underlying respiratory cause.

*Ambient air pollution and meteorology data*. Hourly average PM_2.5_, PM_10_, CO, O_3_, nitrogen dioxide (NO_2_), and sulfur dioxide (SO_2_) were obtained from the Environment Protection Authority (EPA) Victoria (http://www.epa.vic.gov.au) using a central monitoring station in inner suburban Melbourne. Hourly average observations of temperature and relative humidity were obtained from the Bureau of Meteorology monitoring site at Melbourne Airport.

*Fire season and fire-hours.* The fire season for the purposes of this paper was defined as the period from November 2006 through March 2007 because this is the annual fire danger season in Victoria. This period has the highest “fire danger ratings” (which is an indication of how dangerous a fire would be if it started), and the vast majority of days on which total fire bans were declared in Victoria occurred within this period (30 total fire ban days were declared in 2006 and 2007, and only 3 fell outside this period).

“Fire-hours” are defined as the periods when the Melbourne population was most likely to have been affected by forest fire smoke, and they can be identified by a combination of chemical transport modeling and observed increases in particle and gas concentrations during forest fires. Details of the criteria for the identification of fire-hours are presented in Supplemental Material (see Supplemental Material, “Criteria for the identification of fire-hours,” and Supplemental Material, Figures S1 and S2 and Table S1). In summary, fire-hours were identified as those when the hourly PM_2.5_ concentration was > 50 μg/m^3^, the hourly carbon monoxide concentration was > 50 ppm, and the back trajectories for air masses at 1,000 m elevation were in the northwest to northeast sector (315° to 45°) where the forest fires were occurring.

*Statistical analysis*. Case-crossover analysis. A case-crossover analysis was conducted using a time-stratified referent period to select control exposures associated with each index case, where case exposure was the exposure in the hour the OHCA occurred. The reference exposures were the exposures in the day and hour of the case on all days falling within the same month and on the same day of the week as the case. This approach eliminated confounding by hour of the day, day of the week, and monthly trends and also seasonal and long-term trends in the exposure variables (Bateson and Schwartz 1999, 2001; Maclure 1991).

OHCA was the binary outcome (dependent) variable in the analysis. The exposure variables were hourly average pollutant concentrations. Analyses were done for lag 0 (hour of arrest), lag 1 (hour before arrest), lag 2, and so on, and average concentrations of lag 0–2 (average of hour of arrest, lag 1, and lag 2), lag 0–3, lag 0–4, lag 0–8, lag 0–12, lag 0–24, and lag 0–48. In addition, analyses were done using the whole year (from 1 July 2006 through 30 June 2007) and for the fire season only (1 November 2006 through 31 March 2007). Stratification was done by sex, age group (35–64, 65–74, ≥ 75 years), and both age and sex.

Temperature and relative humidity were included as potential confounders. Conditional logistic regression models were used to evaluate the association between the pollutants and OHCA. The parameter estimates from these models may be interpreted as proportional changes in the odds (also referred to as rate in this manuscript), calculated from the odds ratios for the interquartile range (IQR) of the pollutant. The percentage difference in the odds (rate) was calculated from the OR using the formula: (OR – 1) × 100.

As a first step, single-pollutant models were developed, followed by multi-pollutant models, which included those pollutants that showed associations.

Calculation of excess OHCAs attributable to exposure to PM_2.5_ due to forest fire smoke. Using the fire-hour data, we merged the hourly pollution data such that each pollutant recording had a binary variable indicating whether that hour was associated with a forest fire period. We constructed a second binary variable with a default value of 0, but equal to 1 where the current or any of the preceding 47 hr included fire-hours periods. We calculated the total number of hours where at least 1 hr in the preceding 48 hr was associated with forest fire smoke. This represented the risk period.

We used two approaches for estimating the number of OHCA attributable to the forest fires: *a*) a model-derived estimate, using the OR for and IQR increase of PM_2.5_ levels in the 48 hr preceding the arrest, and *b*) a direct calculation based on the difference in the rates between the fire-hours and non–fire-hours. For details of these two approaches, see Supplemental Material, “Methodology: calculation of excess out-of-hospital cardiac arrests.”

All analyses were conducted using Stata (version 12.1; StataCorp, College Station, TX, USA). *p*-Values < 0.05 were considered statistically significant.

## Results

*Study population and exposure description*. A total of 2,046 OHCAs occurred during the study period (July 2006 through June 2007) in metropolitan Melbourne; 64% were men, and the mean age was 71.8 ± 14.2 years. Of these, 783 (38%) occurred during the fire season (November 2006 through March 2007). Men were significantly younger than women when an OHCA occurred, 69.6 versus 75.8 years, respectively (*p* < 0.01).

**Figure 1:**
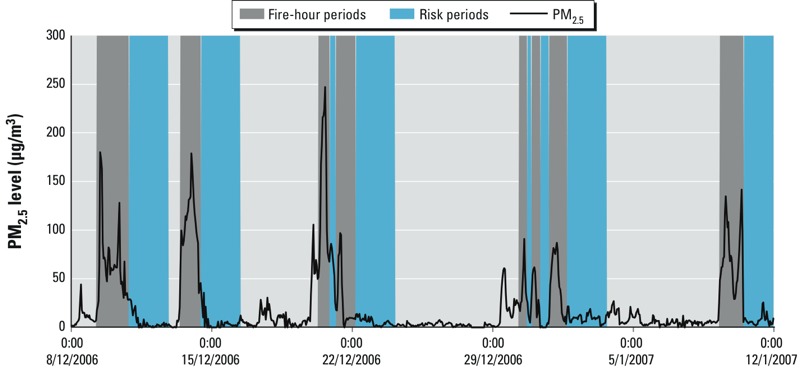
Hourly average PM_2.5_ concentration in Melbourne from 8 December 2006 through 12 January 2007 (0:00, midnight). The dark gray areas represent the “fire-hours” (periods with forest fire smoke), and the blue areas represent the “risk period” (at least 1 fire-hour in the previous 48 hr).

Average hourly concentrations of air pollutants and weather data for the fire season and for the whole year are displayed in Table 1. PM_2.5_ was the pollutant that showed the greatest increase when comparing concentrations between the fire season and non-fire season. Figure 1 presents the hourly PM_2.5_ concentrations in December 2006 and January 2007 and clearly shows the high peak exposures that occurred during exposure to forest fire smoke. The highest hourly PM_2.5_ exposure was 247.2 μg/m^3^, which occurred at 1500 hours on 20 December 2006.

**Table 1:** Average of hourly air pollution and meteorological data for the whole year (1 July 2006–30 June 2007), for the risk period,*^a^* and for the nonrisk period.*^b^*

Exposure	*n*, whole year	Mean, whole year	Mean, risk period	Mean, nonrisk period	Ratio risk/nonrisk period	Percentile cut point, whole year			
						25th	50th	75th	IQR
PM_2.5_ (μg/m^3^)	8,590	7.6	32.4	6.3	5.2	2.4	4.8	8.5	6.1
PM_10_ (μg/m^3^)	8,618	21.0	55.2	19.2	2.9	11.3	16.9	25.0	13.7
CO (ppm)	8,200	0.42	0.51	0.42	1.2	0.2	0.3	0.5	0.3
O_3_ (ppb)	8,201	17.1	33.3	16.2	2.1	6	16	23	17
NO_2_ (ppb)	8,226	11.3	8.8	11.4	0.8	5	9	16	11
SO_2_ (ppb)	8,177	0.84	0.6	0.86	0.7	0	1	1	1
Temperature (ºC)	8,708	14.9	21.6	14.5	1.5	10.2	13.9	18.3	8.1
Relative Humidity (%)	8,708	64.8	45.6	65.8	0.7	50	68	82	32
^***a***^Those hours where at least 1 “fire-hour” occurred in the previous 48 hr. ^***b***^Those hours in the whole year except for the risk period.									

A total of 174 fire-hours were identified during the 2006–2007 fire season, over 12 days (Figure 1). The number of fire-hours per day ranged from 4 to 21 hr. The 12 days fell in the period between 9 December 2006 and 10 January 2007. The average PM_2.5_ concentration during the fire-hours was 106 μg/m^3^.

*Association between air pollution exposure and OHCA*. Table 2 displays the results of a percentage increase in the rate of OHCA for an IQR increase in airborne PM and CO, both for the entire study year (June 2006–July 2007) and for the fire season (November 2006–March 2007). The results for all analyses can be found in Supplemental Material, Table S2. An increased risk of OHCA was observed for an IQR increase in the 48-hr lagged PM_2.5_, both overall (4.4%; 95% CI: 0.2, 8.7%) and among men (7.8%; 95% CI: 2.5, 13.3%). IQR increases in 24-hr and 48-hr CO levels were also associated with increased risks of OHCA among men. During the fire season, the 48-hr estimated effects of PM_2.5_ remained significant, at 5.4% (95% CI: 0.9, 10.1%). In addition, the 48-hr IQR increase in CO became significantly associated with the risk of OHCA (10.0%; 95% CI: 0.6, 20.2%). Among men in the fire season, greater increases in OHCA were observed with IQR increases in the 48-hr lagged PM_2.5_, PM_10_, and CO. There was no significant association between the risk of OHCA and any of the air pollutants among women.

**Table 2:** Estimated percentage difference (95% CI) in the rate of out-of-hospital cardiac arrest for an IQR increase in each air pollutant*^a^* using conditional logistic regression models.*^b^*^,^*^c^*

Study population	Hourly lags	PM_2.5_	PM_10_	CO
Whole year (July 2006–June 2007)
Total (*n* = 2,046)	0	1.3 (–1.0, 3.8)	–0.2 (–4.1, 3.8)	–0.6 (–4.8, 3.9)
	0–24	3.0 (–0.3, 6.5)	3.9 (–1.5, 9.6)	2.7 (–3.3, 9.2)
	0–48	4.4 (0.2, 8.7)*	4.0 (–2.4, 10.8)	5.6 (–1.6, 13.2)
Men (*n *= 1,311)	0	2.2 (–0.7, 5.3)	1.4 (–3.5, 6.5)	1.1 (–4.3, 6.9)
	0–24	4.9 (0.7, 9.3)*	6.6 (–0.2, 13.9)	8.0 (0.1, 16.6)*
	0–48	7.8 (2.5, 13.3)**	8.4 (0.1, 17.3)*	10.0 (0.6, 20.2)*
Women (*n* = 735)	0	–0.2 (–4.2, 3.9)	–2.9 (–9.3, 3.9)	–4.1 (0.6, 20.2)
	0–24	–0.4 (–6.0, 5.5)	–0.9 (–9.6, 8.6)	–6.0 (–15.1, 4.1)
	0–48	–1.8 (–8.6, 5.4)	–3.4 (–13.3, 7.7)	–1.4 (–15.1, 4.1)
Fire season (November 2006–March 2007)
Total (*n* = 783)	0	1.9 (–0.6, 4.5)	3.0 (–1.4, 7.5)	3.9 (–6.0, 14.8)
	0–24	3.5 (–0.1, 7.3)	7.0 (0.8, 13.6)*	16.5 (–0.1, 35.8)
	0–48	5.4 (0.9, 10.2)*	7.7 (0.3, 15.8)*	24.6 (4.5, 48.0)*
Men (*n* = 500)	0	2.5 (–0.7, 5.7)	4.5 (–1.0, 10.3)	6.9 (–5.8, 21.3)
	0–24	4.7 (0.1, 9.4)*	8.3 (0.6, 16.6)*	24.6 (2.9, 50.8)*
	0–48	8.1 (2.3, 14.1)**	11.1 (1.6, 21.5)*	35.7 (9.0, 68.9)**
Women (*n* = 283)	0	0.9 (–3.3, 5.2)	0.4 (1.5, 21.5)	–1.3 (–16.2, 16.2)
	0–24	1.1 (–4.9, 7.6)	4.1 (–6.0, 15.3)	0.9 (–22.4, 31.4)
	0–48	0.2 (–7.2, 8.2)	1.3 (–10.4, 14.4)	4.3 (–22.1, 39.8)
^***a***^IQR is based on the distribution of the whole year. IQRs are as follows: 6.1 μg/m^3^ (PM_2.5_), 13.7 μg/m^3^ (PM_10_), and 0.3 ppm for CO. ^***b***^No significant results for less than 24-hr rolling average. ^***c***^Adjusted for temperature and relative humidity. *p < 0.05. **p < 0.01.				

The hour of the reported arrest and the IQR change in O_3_ for the whole year lagged 2 and 4 hr were associated with increases in OHCA in 65- to 74-year-olds for the entire duration of the study (Table 3). During the fire season period, similar results for O_3_ and OHCA were observed among 65- to 74-year-olds, although the confidence intervals were much wider. In addition, among those > 75 years old, 8- and 12-hr lags were associated with an increased risk. For women, 8- and 12-hr lags of an IQR change in O_3_ were associated with OHCA during the fire season.

**Table 3:** Estimated percentage difference in the rate of out-of-hospital cardiac arrest for an IQR increase in O_3_*^a^* of 17 ppb using conditional logistic regression models.

Hourly lags	Age group (years)				Sex	
	≥ 35	35–64	65–74	≥ 75	Men	Women
Whole year (1 July 2006– 30 June 2007)
0	5.9 (–4.6, 17.5)	–7.9 (–23.2, 10.3)	43.8 (10.8, 86.5)**	7.5 (–7.6, 25.0)	6.5 (–6.6, 21.3)	6.8 (–10.3, 27.0)
0–2	4.3 (–6.1, 15.9)	–10.5 (–25.6, 7.7)	42.8 (10.4, 84.8)**	5.6 (–9.3, 23.0)	4.4 (–8.5, 19.2)	5.7 (–11.1, 25.7)
0–4	3.4 (–7.0, 15.1)	–10.7 (–26.2, 8.2)	36.1 (5.2, 76.0)*	4.9 (–10.0, 22.2)	3.9 (–9.1, 18.8)	4.2 (–12.8, 24.5)
0–8	6.9 (–4.2, 19.2)	–5.4 (–22.3, 15.2)	25.2 (–3.6, 62.6)	10.5 (–5.2, 28.8)	3.8 (–9.4, 19.0)	13.9 (–5.2, 36.8)
0–12	7.9 (–4.1, 21.3)	–5.5 (–23.6, 16.9)	20.0 (–8.5, 57.1)	13.9 (–3.5, 34.4)	3.6 (–10.5, 19.9)	17.1 (–3.8, 42.4)
0–24	4.3 (–8.7, 19.2)	–6.8 (–26.9, 18.9)	23.7 (–9.1, 68.4)	7.3 (–11.0, 29.3)	1.8 (–13.8, 20.2)	9.6 (–12.3, 36.9)
0–48	–0.3 (–14.2, 15.9)	–1.8 (–25.0, 28.5)	2.4 (–28.2, 46.2)	0.6 (–18.6, 24.3)	0.7 (–16.5, 21.5)	–2.5 (–24.2, 25.4)
Fire season (1 November 2006–31 March 2007)
0	9.1 (–4.7, 25.0)	2.4 (–17.6, 27.3)	48.9 (2.4, 116.6)*	8.5 (–11.4, 33.0)	5.4 (–11.2, 25.2)	17.8 (–5.8, 47.3)
0–2	8.6 (–5.3, 24.5)	–2.5 (–22.3, 22.4)	57.0 (8.3, 127.6)*	9.3 (–10.8, 33.9)	4.7 (–12.1, 24.7)	17.3 (–5.9, 46.3)
0–4	11.5 (–3.0, 28.0)	0.1 (–20.9, 26.8)	56.2 (8.3, 125.3)*	11.8 (–8.6, 36.9)	7.9 (–9.6, 28.7)	19.4 (–4.7, 49.7)
0–8	20.0 (3.8, 38.6)	9.6 (–14.6, 40.5)	45.5 (0.7, 110.3)*	23.4 (0.5, 51.5)*	11.2 (–7.2, 33.4)	38.8 (9.0, 76.7)**
0–12	24.1 (5.4, 46.2)	12.0 (–15.5, 48.4)	20.9 (–18.1, 78.5)	38.1 (9.0, 75.1)**	12.4 (–8.5, 38.1)	49.0 (13.4, 95.7)**
0–24	15.2 (–5.2, 40.1)	14.8 (–18.2, 61.1)	–8.7 (–43.2, 46.8)	30.3 (–1.4, 72.2)	5.9 (–17.2, 35.5)	33.7 (–3.2, 84.6)
0–48	8.1 (–12.5, 33.5)	21.2 (–15.3, 73.4)	–25.9 (–57.0, 27.8)	13.9 (–15.6, 53.7)	4.8 (–19.5, 36.5)	14.1 (–19.8, 62.3)
^***a***^Adjusted for temperature and relative humidity. *p < 0.05. **p < 0.01.						

Two-pollutant models were developed for 48-hr PM_2.5_ with O_3_ and CO. For O_3_, both the 48-hr lag and the 2-hr lags were investigated (because significant associations were seen in the O_3_ single-pollutant model for the 2-hr lag), and PM_2.5_ remained significant (4.7%; 95% CI: 0.4, 9.3% and 7.5%; 95% CI: 2.0, 13.3, respectively). However, O_3_ became nonsignificant (–4.7%; 95% CI: –18.4, 11.3% and 2.5%; 95% CI: –10.2, 17.0%, respectively). The two-pollutant models for PM_2.5_ and CO (48-hr average) resulted in both associations becoming nonsignificant (3.71%; 95% CI: –1.2, 8.9% and 2.0%; 95% CI –6.3, 10.9%, respectively). However, restricting this analysis to males only resulted in a significant association with PM_2.5_ but not CO (6.7%; 95% CI: 0.5, 13.3% and 3.3%; 95% CI: –7.11, 14.9, respectively). The correlation between PM_2.5_ and O_3_ was 0.24, and with PM_2.5_ and CO was 0.37.

*Excess arrests attributable to forest fire smoke*. Two methods were used to estimate the number of excess arrests that were attributable to forest fire smoke (see Supplemental Material, “Methodology: calculation of excess out-of-hospital cardiac arrests”). Using the model-derived calculation, we estimated that 23.9 (95% CI: 3.1, 40.2) excess arrests were associated with the fire-hours; and using the direct calculation, we estimated that 28.9 (95% CI: 3.8, 52.9) excess arrests were associated with the fire-hours.

The mean temperature was higher in the hours that were identified as being affected by forest fire smoke when compared with the other hours throughout the year (21.65 vs. 14.53°C). However, each degree increase in temperature was associated with only a 0.02% increase in risk of OHCA, and after adjusting for this there was no change in the estimated number of excess arrests.

## Discussion

This study shows that exposure to forest fire smoke in the 2006–2007 Victoria fire season was associated with an increased risk of having an OHCA in Melbourne, its capital city with a population of about 4 million. In addition, 24–29 excess OHCAs were estimated to have occurred in Melbourne because the air quality was affected by smoke from the forest fires.

Although hourly air quality data were available, and several short-term averages (including 1, 2, 4, 8, and 12 hr) were investigated, the strongest association was found with the 24-hr and 48-hr rolling averages for PM_2.5_ and CO before the OHCA occurred, suggesting that there was a delayed or cumulative association. This association was seen predominantly in men. We are not aware of any plausible reasons why the associations were seen particularly in men, and did not have data concerning recognized cardiac risk factors for coronary artery disease. However, almost two-thirds of the OHCA during the 2006–2007 fire season occurred in men, and men having OHCAs were significantly younger than women. Our observations could possibly reflect only the higher age-related incidence of coronary artery disease and OHCA and effects on the background of greater abnormalities of other risk factors in men. Perhaps it is related to confounding factors such as smoking or blood pressure, or even other factors such as behavioral differences (e.g., time spent outside could play a role). We are aware of no other studies to date that have investigated OHCA and PM_2.5_ during forest fire smoke events. However, of the studies investigating urban PM_2.5_ and OHCA, some have reported a strong association for men (Dennekamp et al. 2010; Ensor et al. 2013) whereas others did not find this (Rosenthal et al. 2008; Silverman et al. 2010; Sullivan et al. 2003). A study from Launceston, Australia, looking at cardiovascular mortality found similar sex-specific observations. The air pollution there derived mainly from biomass combustion (residential woodsmoke), but the authors found a significant improvement in cardiovascular mortality when air pollution decreased, though this was not found in women (Johnston et al. 2013).

For O_3_, when we analyzed all OHCAs, the coefficients were not significantly elevated. Only when we stratified by age and sex were significant positive associations observed, and only at rolling averages of ≤ 12 hr. In the literature, the studies investigating the association between urban O_3_ and OHCA show inconsistent results. Some did not find associations (Dennekamp and Abramson 2011; Silverman et al. 2010) and some very recent studies did (Ensor et al. 2013; Raza et al. 2014; Rosenthal et al. 2013). Of the latter, one study found statistical associations with O_3_ exposure 2 hr, 24 hr, and 72 hr before an OHCA (Raza et al. 2014); another found significant associations only with lag 2 days exposures and not hourly lagged exposures (Rosenthal et al. 2013); and another found associations with both hourly and daily (maximum 8-hr average) exposure (Ensor et al. 2013; Raza et al. 2014; Rosenthal et al. 2013).

The PM_2.5_ associations found here are relatively similar to those of our previous study, in which we investigated the association between urban air pollution and OHCA in Melbourne (Dennekamp et al. 2010). A 4.25-μg/m^3^ increase in PM_2.5_ was associated with a a 3.61% increase in risk of OHCA (95% CI: 1.29, 5.99%) in our urban air pollution study, and of 3.75% (95% CI: 0.60, 7.00%) during the fire season in the present study. However, even though the estimated effects may be similar, the change in air quality concentrations is much larger during forest fire episodes compared with non–forest fire episodes, hence resulting in a measureable excess in OHCAs during relatively short smoke episodes.

The estimated effects for PM_2.5_, PM_10_, O_3_, and CO were almost all larger and stronger in the fire season despite the smaller sample size. This association was confirmed by the analysis using fire-hours, which showed a significant increase in the number of OHCAs.

Our findings suggest that PM_2.5_ seems to be the key pollutant associated with excess OHCA during forest fires. First of all, in our study PM_2.5_ increases the most during a forest fire season compared with any of the other pollutants that were monitored (including PM_10_). And even with introduction of O_3_ and CO, the PM_2.5_ association remained consistent. However, this should be interpreted with caution because of the high correlation between the pollutants, particularly between PM_2.5_ and CO (*r* = 0.37).

Previous studies have not shown consistent associations between exposure to forest fire smoke and cardiovascular outcomes (Delfino et al. 2009; Hanigan et al. 2008; Henderson et al. 2011; Johnston et al. 2007, 2011; Martin et al. 2013; Morgan et al. 2010; Mott et al. 2005; Schranz et al. 2010). Most of these studies investigated hospital admissions for cardiovascular disease in general or for a specific disease type (e.g., myocardial infarction), whereas ours, to our knowledge, is the first study to clearly show an association between ambulance data for OHCA and forest fire smoke. Perhaps we find an association here because the vast majority of OHCAs are fatal, and therefore these cases do not appear in hospital emergency presentations or hospital admission records. From July 2006 through June 2007 the proportion of OHCA attended by ambulance in Melbourne (≥ 35 years of age and presumed cardiac) who were declared deceased at the scene and not transported to hospital was 78.4%. The severe outcome of arrest that could be associated with air pollution would result in a selection bias for studies that relied on hospital admissions.

Although no studies have previously investigated the association between OHCA and forest fire smoke, several studies have investigated urban PM_2.5_ and OHCA, with inconsistent findings. No significant associations were found in some (Levy et al. 2001; Raza et al. 2014; Sullivan et al. 2003); another study found null results overall except for a subgroup that had arrests that were witnessed by bystanders (Rosenthal et al. 2008); and a few large studies did find associations between urban PM_2.5_ and OHCAs (Dennekamp et al. 2010; Ensor et al. 2013; Silverman et al. 2010). Even though the present study was rather small, it is likely we found a significant association due to the large PM_2.5_ concentrations that occur during a forest fire season, and hence we were able to detect a significant increase in excess OHCAs.

Our data could not provide further insights into the underlying mechanisms involved. However, these have been extensively reviewed elsewhere (Brook et al. 2010). We have hypothesized that PM exposure may cause systemic inflammation that can lead to an increase in blood coagulability with resultant coronary thrombosis (Seaton and Dennekamp 2003). In addition, the risk of potentially lethal cardiac arrhythmias and cardiac arrest may be increased, possibly partly because of impaired cardiac autonomic control mechanisms (Brook et al. 2010; Luttmann-Gibson et al. 2010). However, this hypothesis is not supported by two large studies in patients with implantable defibrillators; the authors did not find associations between PM levels and tachyarrhytmic events (Anderson et al. 2010; Metzger et al. 2007). Studies have shown O_3_ exposure to be associated with disturbed heart rate variability (Kop et al. 2001; Park et al. 2005; Utell et al. 2002).

*Strengths and limitations*. A major strength of this study is the health outcome data. The Victorian Ambulance Cardiac Arrest Registry is comprehensive, covering close to 100% of the OHCAs that occur in Melbourne and are attended by ambulance.

A limitation of this study (and with most other air pollution studies) is the use of one central monitoring location in Melbourne for the air pollutant concentrations that was used to represent exposure for the whole of metropolitan Melbourne. At the time of the study there were only two EPA Victoria monitors measuring PM_2.5_, both in inner Melbourne. One of the sites has a significant number of data missing during the study period, whereas the other site had near complete data for all pollutants. Where data were available, we found that the correlation between the two monitors was very high (*R* = 0.95), and therefore the data from one monitoring station were used in this study. The exposure misclassification resulting from this is likely to underestimate the association and the number of attributable arrests. The fire-hour data were derived from a location 30 km from the EPA monitoring station. This would explain the fact that Figure 1 shows that on two occasions the concentration increases sharply and the fire-hour starts a couple of hours later. However, considering our analysis used the 48 hr before an OHCA, this is unlikely to have a measurable effect on our results. Modeled data that would more accurately reflect probable exposure in areas that do not have monitoring are recommended for future studies.

Another limitation is that it is not possible to draw conclusions regarding susceptible subgroups in the community, because we do not have detailed information on individual risk factors and co-morbidities. This is not a problem statistically, because in a case-crossover design these factors are adjusted for by design. However, the utility of the findings are reduced as advice can be provided only in general terms, rather than being specific to different risk groups.

In conclusion, the results suggest that exposure to forest fire smoke is associated with the occurrence of out-of-hospital cardiac arrests in men. It is estimated that in the 2006–2007 forest fire season the smoke was responsible for 24–29 excess arrests in Melbourne. The impact of this is likely to increase in the future, because forest fires are likely to increase in frequency and severity in many countries where forest fires occur in close proximity to large population centers. These findings have implications for public health messages to raise community awareness and for planning of emergency services during forest fire seasons.

## Supplemental Material

(865 KB) PDF
