# Supplementary material for: Forest Fire Smoke Exposures and Out-of-Hospital Cardiac Arrests in Melbourne, Australia: A Case-Crossover Study
Source: Environ Health Perspect. 2015 Mar 20;123(10):959–64. doi: 10.1289/ehp.1408436 (PMC4590745; doi:10.1289/ehp.1408436)
Supplement: (865 KB) PDF [file ehp.1408436.s001.acco.pdf]

*Environ Health Perspect*
doi: 10.1289/ehp.1408436

**Note to Readers:** ***EHP*** **strives to ensure that all journal content is accessible to all readers. However, some figures and Supplemental Material published in** ***EHP*** **articles may not conform to 508 standards due to the complexity of the information being presented. If you need assistance accessing journal content, please contact ehp508@niehs.nih.gov. Our staff will work with you to assess and meet your accessibility needs within 3 working days.**

# Supplemental Material

# Forest Fire Smoke Exposures and Out-of-Hospital Cardiac Arrests in Melbourne, Australia: A Case-Crossover Study

Martine Dennekamp, Lahn D. Straney, Bircan Erbas, Michael J. Abramson, Melita Keywood, Karen Smith, Malcolm R. Sim, Deborah C. Glass, Anthony Del Monaco, Anjali Haikerwal, and Andrew M. Tonkin

## Table of Contents

1. Criteria for the identification of fire-hours

**Figure S1.** Time series of hourly concentrations of $PM_{2.5}$ and CO. Filled in circles represent fire periods, lines represent concentration of $PM_{2.5}$ in the top figure and CO in the bottom figure.

**Figure S2.** Hourly $PM_{2.5}$ and CO concentrations as a function of wind direction. Diameter of the circle represents concentration (also represented by the vertical axis with the scale and units i.e. for the left diagram, the diameter of the circle represents PM2.5 concentration of 30 μg $m^{-3}$ from the centre of the circle to the circumference of the circle; for the right hand diagram the diameter of the circle represents CO concentration of 450 ppb from the centre of the circle to the circumference of the circle. North is represented by 0 on the circumference of the circle east by 90, south by 180 and west by 270.

**Table S1.** Selection criteria for smoke impacted periods.

2. Methodology - calculation of excess out-of-hospital cardiac arrests

*1. Model-derived calculation*

*2. Direction calculation*

**Table S2.** Estimated percentage difference in the relative odds of out-of-hospital cardiac arrest for an interquartile range increase in individual pollutants based on conditional logistic regression models adjusted for temperature and relative humidity. Single Pollutant models by age, sex and non-fire vs fire period.

1. Criteria for the identification of fire-hours

**(By Melita Keywood - CSIRO Marine and Atmospheric Research, Aspendale, Victoria, Australia)**

Bushfire smoke impacted periods were identified by a combination of increases in particle and gas concentrations and wind directions. Figure S1 shows the time series of hourly concentrations of $PM_{2.5}$ and CO for the period 1 November 2006 to 28 February 2007. The time series are marked by significantly high concentrations in most of the $PM_{2.5}$ and CO between mid December and early January. Figure S2 also reveals that in general high concentrations of a $PM_{2.5}$ and CO is associated with wind directions from the east and north east as is the concentration of CO.

The above observations were used to derive criteria with which to select periods impacted by bushfire smoke so that the contribution of bushfire smoke to air quality at the Aspendale site could be compared with air quality under non-bushfire effected summer conditions. The criteria used to select the bushfire impacted periods are listed in Table S1.

$PM_{2.5}$ was measured every 5 minutes using a Tapered Element Oscillating Microbalance Filter Dynamic Measurement System (TEOM 8500 FDMS Rupprecht & Patashnik, Albany NY). CO was measured by infra red photometric detection gas filter correlation spectroscopy (Ecotech EC9830T CO trace analyser, Knoxfield, Australia).

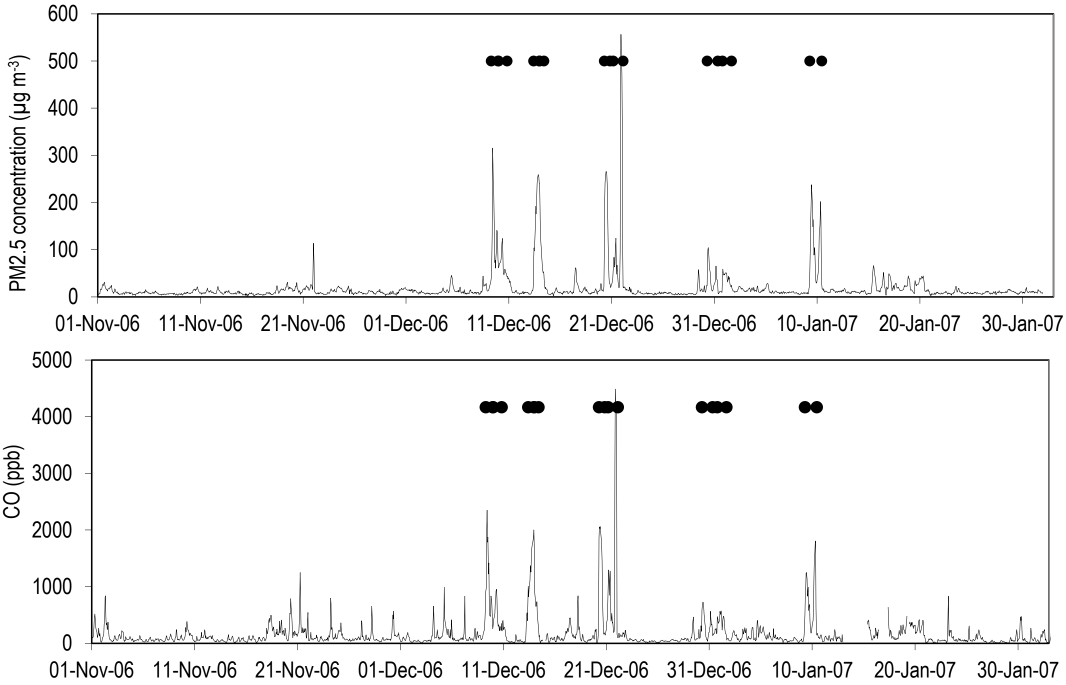


**Figure S1.** Time series of hourly concentrations of $PM_{2.5}$ and CO. Filled in circles represent fire periods, lines represent concentration of $PM_{2.5}$ in the top figure and CO in the bottom figure.

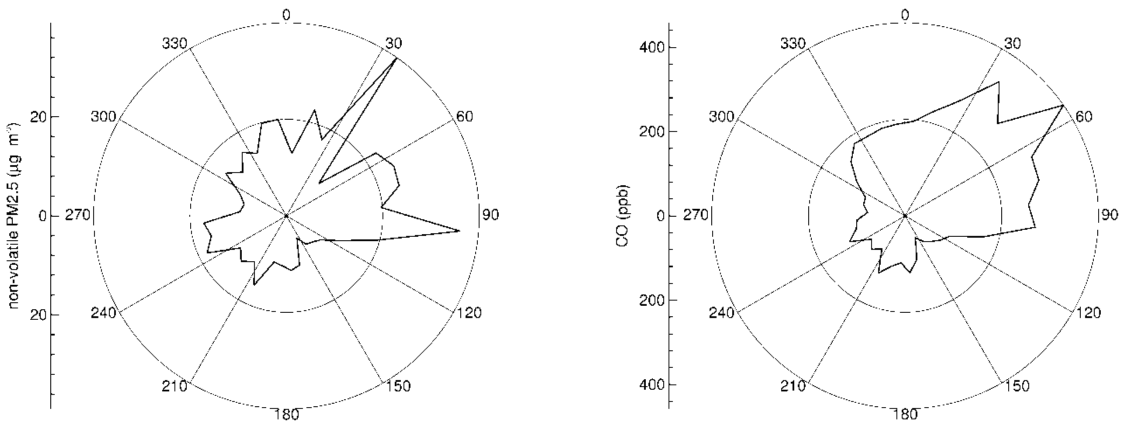


**Figure S2.** Hourly $PM_{2.5}$ and CO concentrations as a function of wind direction. Diameter of the circle represents concentration (also represented by the vertical axis with the scale and units i.e. for the left diagram, the diameter of the circle represents $PM_{2.5}$ concentration of 30 µg $m^{-3}$ from the centre of the circle to the circumference of the circle; for the right hand diagram the diameter of the circle represents CO concentration of 450 ppb from the centre of the circle to the circumference of the circle. North is represented by 0 on the circumference of the circle east by 90, south by 180 and west by 270.

**Table S1.** Selection criteria for smoke impacted periods.

| **Criteria (hourly data)** |
|---|
| $PM_{2.5}$ concentration > 50 µg/$m^3$ |
| CO concentration > 50 ppb |
| Wind direction 1000 m trajectory origin 315-45° |

## 2. Methodology - calculation of excess out-of-hospital cardiac arrests

We describe two approaches for estimating the number of arrests attributable to the forest fires. First, a model derived estimate, using the odds ratio for an interquartile range increase of $PM_{2.5}$ levels in the 48 hours preceding the arrest, and second a direct calculation based on the difference in the rates between the ‘fire-hours and non-‘fire-hours’.

### *1. Model-derived calculation*

We estimated the excess number of arrests associated with increases in the 48 hour average of $PM_{2.5}$ during the Risk periods. We can estimate the excess risk as follows, where *P* represents the mean 48 hour average of PM2.5 and the subscripts 1 and 2 are used to represent the Risk and Non-Risk periods respectively. Thus the number of interquartile range increases in the mean value can be calculated as $(P_1-P_2)/IQR$.

Recall that the odds ratio approximates the relative risk where the effect size is small and the initial risk low. Using the odds ratio, OR, for the 48 hour average of $PM_{2.5}$ and letting a represent the number of arrests, we estimate the excess risk ER as $ER = [(P_1 – P_2)/IQR](OR – 1)$.

To propagate the uncertainty of the model coefficient in our estimate, we drew 1000 values for the odds ratio using the normal distribution and standard error of the parameter. We provide a 95% uncertainty interval which is given by the $2.5^{th}$ and $97.5^{th}$ percentile values of the 1000 estimates. From this we derived 1000 estimates of the ER and the subsequent excess arrests, E, which is calculated as $E = a_1 – [a_1/(1 + ER)]$, where $a_1$ represents the number of arrests in the Risk period.

***2. Direction calculation***

The rate difference was estimated as follows, where a represents the number of arrests, t represents the time at risk and n represents the number of people at risk. We use the subscripts 1 and 2 to represent the Risk and Non-Risk periods respectively. Thus, the rate difference can be calculated as $[a_1/(t_1n_1)]– [a_2/(t_2n_2)]$.

If we assume that the number of people at risk remained constant throughout the year, we can rearrange, such that $1/n[(a_1/t_{1)}– (a_2/t_2)]$

The excess arrests E are given by the rate difference multiplied by the person time at risk in the Risk period. Thus, $E = 1/n\ [(a_1/t_{1)}– (a_2/t_2)]nt_1$

Simplifying, $E = [(a_1/t_{1)}– (a_2/t_2)]t_1$

To reflect the uncertainty in the observed rates, we drew 1000 random values for $a_1$ and $a_2$ using separate Poisson distributions where the mean value was given by the observed.

The direct calculation did not adjust for ambient temperature which is known to be associated with increased risk of out-of-hospital cardiac arrest. It is reasonable to expect that the estimate derived from the direct calculation might overestimate the number of attributable OHCA's and therefore we used the odds ratio for temperature estimated in the model for the 48 hour average of $PM_{2.5}$ to adjust the estimate.

**Table S2.** Estimated percentage difference in the relative odds of out-of-hospital cardiac arrest for an interquartile range increase in individual pollutants based on conditional logistic regression models adjusted for temperature and relative humidity. Single Pollutant models by age, sex and non-fire vs fire period.

| Period[a] | Stratification | Lag (hours) | $PM_{2.5}$ % difference | (95% CI) | | $PM_{10}$ % difference | (95% CI) | CO % % difference | (95% CI) | |
|---|---|---|---|---|---|---|---|---|---|---|
| All data (07/06 to 06/07) | All data | 0 | 1.35 | (-1.02, 3.77) | | -0.19 | (-4.06, 3.83) | -0.58 | (-4.84, 3.87) | |
| All data (07/06 to 06/07) | All data | 1 | 1.21 | (-1.23, 3.71) | | 1.45 | (-2.49, 5.55) | -0.38 | (-4.72, 4.15) | |
| All data (07/06 to 06/07) | All data | 2 | 0.13 | (-2.36, 2.68) | | -0.39 | (-4.34, 3.73) | 0.40 | (-3.93, 4.93) | |
| All data (07/06 to 06/07) | All data | 0-2 | 1.30 | (-1.15, 3.81) | | 0.54 | (-3.50, 4.75) | -0.16 | (-4.54, 4.42) | |
| All data (07/06 to 06/07) | All data | 0-3 | 0.94 | (-1.58, 3.53) | | 0.05 | (-4.10, 4.39) | -0.07 | (-4.61, 4.68) | |
| All data (07/06 to 06/07) | All data | 0-4 | 0.93 | (-1.66, 3.59) | | 0.41 | (-3.87, 4.87) | -0.30 | (-5.02, 4.65) | |
| All data (07/06 to 06/07) | All data | 0-8 | 1.74 | (-1.04, 4.59) | | 2.57 | (-2.09, 7.46) | 1.39 | (-3.94, 7.03) | |
| All data (07/06 to 06/07) | All data | 0-12 | 1.84 | (-1.05, 4.82) | | 2.81 | (-2.02, 7.88) | 0.97 | (-4.33, 6.57) | |
| All data (07/06 to 06/07) | All data | 0-24 | 3.03 | (-0.32, 6.50) | * | 3.90 | (-1.49, 9.58) | 2.74 | (-3.32, 9.19) | |
| All data (07/06 to 06/07) | All data | 0-48 | 4.36 | (0.20, 8.69) | ** | 4.02 | (-2.39, 10.84) | 5.55 | (-1.60, 13.23) | |
| All data (07/06 to 06/07) | 35-64 years | 0 | 1.18 | (-2.38, 4.87) | | -0.07 | (-6.05, 6.29) | -0.83 | (-8.27, 7.22) | |
| All data (07/06 to 06/07) | 35-64 years | 1 | 0.73 | (-2.88, 4.47) | | 0.48 | (-5.51, 6.85) | -0.85 | (-8.96, 7.98) | |
| All data (07/06 to 06/07) | 35-64 years | 2 | -0.47 | (-4.18, 3.38) | | -1.13 | (-7.31, 5.46) | -3.01 | (-11.18, 5.91) | |
| All data (07/06 to 06/07) | 35-64 years | 0-2 | 1.05 | (-2.60, 4.83) | | 0.12 | (-6.03, 6.68) | 0.05 | (-7.90, 8.70) | |
| All data (07/06 to 06/07) | 35-64 years | 0-3 | 0.60 | (-3.14, 4.47) | | -0.59 | (-6.92, 6.16) | -0.79 | (-9.14, 8.32) | |
| All data (07/06 to 06/07) | 35-64 years | 0-4 | 0.33 | (-3.51, 4.32) | | -0.47 | (-6.96, 6.47) | -1.01 | (-9.80, 8.64) | |
| All data (07/06 to 06/07) | 35-64 years | 0-8 | 0.49 | (-3.70, 4.86) | | 0.35 | (-6.73, 7.96) | 2.70 | (-7.62, 14.19) | |
| All data (07/06 to 06/07) | 35-64 years | 0-12 | 1.75 | (-2.75, 6.45) | | 2.12 | (-5.51, 10.36) | 5.02 | (-5.47, 16.67) | |
| All data (07/06 to 06/07) | 35-64 years | 0-24 | 5.35 | (-0.25, 11.26) | * | 7.32 | (-1.91, 17.41) | 10.82 | (-1.21, 24.31) | * |
| All data (07/06 to 06/07) | 35-64 years | 0-48 | 7.80 | (0.29, 15.86) | ** | 9.23 | (-2.60, 22.49) | 17.60 | (2.87, 34.45) | ** |
| All data (07/06 to 06/07) | 65-74 years | 0 | 4.14 | (-2.07, 10.73) | | 0.77 | (-8.94, 11.51) | 3.51 | (-6.15, 14.17) | |
| All data (07/06 to 06/07) | 65-74 years | 1 | 3.22 | (-3.04, 9.88) | | 2.69 | (-7.12, 13.55) | 1.44 | (-8.09, 11.97) | |

| Period[a] | Stratification | Lag (hours) | $PM_{2.5}$ % difference (95% CI) | $PM_{10}$ % difference (95% CI) | CO % % difference (95% CI) |
|---|---|---|---|---|---|
| All data (07/06 to 06/07) | 65-74 years | 2 | 2.72 (-3.66, 9.52) | 4.56 (-5.37, 15.53) | 5.30 (-4.50, 16.10) |
| All data (07/06 to 06/07) | 65-74 years | 0-2 | 3.84 (-2.60, 10.72) | 2.05 (-8.14, 13.38) | 4.37 (-5.54, 15.32) |
| All data (07/06 to 06/07) | 65-74 years | 0-3 | 3.71 (-2.92, 10.80) | 3.06 (-7.49, 14.82) | 4.39 (-5.84, 15.73) |
| All data (07/06 to 06/07) | 65-74 years | 0-4 | 3.87 (-2.92, 11.13) | 4.38 (-6.58, 16.62) | 4.09 (-6.41, 15.77) |
| All data (07/06 to 06/07) | 65-74 years | 0-8 | 4.93 (-2.17, 12.55) | 6.07 (-5.64, 19.23) | 3.59 (-7.68, 16.22) |
| All data (07/06 to 06/07) | 65-74 years | 0-12 | 4.45 (-2.71, 12.13) | 4.45 (-7.06, 17.38) | -0.70 (-11.59, 11.53) |
| All data (07/06 to 06/07) | 65-74 years | 0-24 | 4.34 (-3.80, 13.17) | 1.83 (-10.50, 15.85) | -3.80 (-16.16, 10.39) |
| All data (07/06 to 06/07) | 65-74 years | 0-48 | 3.36 (-6.62, 14.41) | -2.21 (-16.38, 14.35) | -2.31 (-16.64, 14.49) |
| All data (07/06 to 06/07) | 75+ years | 0 | 0.54 (-3.12, 4.35) | -0.76 (-6.55, 5.39) | -2.60 (-8.60, 3.80) |
| All data (07/06 to 06/07) | 75+ years | 1 | 0.77 (-3.09, 4.78) | 1.76 (-4.28, 8.19) | -1.59 (-7.53, 4.73) |
| All data (07/06 to 06/07) | 75+ years | 2 | -0.29 (-4.21, 3.80) | -1.54 (-7.45, 4.75) | -0.14 (-6.02, 6.10) |
| All data (07/06 to 06/07) | 75+ years | 0-2 | 0.57 (-3.26, 4.56) | 0.27 (-5.86, 6.79) | -2.70 (-8.73, 3.73) |
| All data (07/06 to 06/07) | 75+ years | 0-3 | 0.24 (-3.73, 4.38) | -0.50 (-6.82, 6.23) | -2.00 (-8.21, 4.63) |
| All data (07/06 to 06/07) | 75+ years | 0-4 | 0.43 (-3.65, 4.69) | -0.26 (-6.76, 6.70) | -2.14 (-8.57, 4.75) |
| All data (07/06 to 06/07) | 75+ years | 0-8 | 1.77 (-2.55, 6.28) | 3.52 (-3.58, 11.13) | -0.59 (-7.85, 7.24) |
| All data (07/06 to 06/07) | 75+ years | 0-12 | 0.88 (-3.54, 5.50) | 2.97 (-4.23, 10.71) | -0.74 (-7.96, 7.05) |
| All data (07/06 to 06/07) | 75+ years | 0-24 | 0.65 (-4.20, 5.73) | 2.31 (-5.34, 10.57) | 0.39 (-7.80, 9.31) |
| All data (07/06 to 06/07) | 75+ years | 0-48 | 2.60 (-3.05, 8.59) | 3.19 (-5.51, 12.69) | 2.37 (-7.15, 12.86) |
| All data (07/06 to 06/07) | Male | 0 | 2.23 (-0.73, 5.29) | 1.38 (-3.48, 6.48) | 1.14 (-4.30, 6.90) |
| All data (07/06 to 06/07) | Male | 1 | 2.63 (-0.40, 5.76) * | 2.58 (-2.36, 7.78) | 2.04 (-3.54, 7.95) |
| All data (07/06 to 06/07) | Male | 2 | 0.96 (-2.09, 4.11) | 1.05 (-3.92, 6.27) | 3.26 (-2.36, 9.20) |
| All data (07/06 to 06/07) | Male | 0-2 | 2.47 (-0.60, 5.63) | 1.88 (-3.20, 7.22) | 2.40 (-3.21, 8.33) |
| All data (07/06 to 06/07) | Male | 0-3 | 2.06 (-1.08, 5.29) | 1.50 (-3.72, 7.00) | 2.86 (-2.97, 9.04) |
| All data (07/06 to 06/07) | Male | 0-4 | 2.01 (-1.22, 5.34) | 1.91 (-3.48, 7.59) | 2.88 (-3.16, 9.30) |
| All data (07/06 to 06/07) | Male | 0-8 | 2.54 (-0.90, 6.10) | 4.05 (-1.79, 10.24) | 6.30 (-0.64, 13.71) * |
| All data (07/06 to 06/07) | Male | 0-12 | 2.74 (-0.87, 6.47) | 4.36 (-1.72, 10.82) | 4.91 (-1.98, 12.29) |

| Period[a] | Stratification | Lag (hours) | $PM_{2.5}$ % difference (95% CI) | | | $PM_{10}$ % difference (95% CI) | | | CO % % difference (95% CI) | | |
|---|---|---|---|---|---|---|---|---|---|---|---|
| All data (07/06 to 06/07) | Male | 0-24 | 4.89 | (0.69, 9.27) | ** | 6.61 | (-0.18, 13.86) | * | 7.99 | (0.06, 16.55) | ** |
| All data (07/06 to 06/07) | Male | 0-48 | 7.76 | (2.48, 13.31) | # | 8.36 | (0.12, 17.27) | ** | 9.96 | (0.61, 20.18) | ** |
| All data (07/06 to 06/07) | Female | 0 | -0.22 | (-4.17, 3.91) | | -2.91 | (-9.26, 3.89) | | -4.07 | (-10.80, 3.17) | |
| All data (07/06 to 06/07) | Female | 1 | -1.38 | (-5.57, 3.00) | | -0.41 | (-6.85, 6.46) | | -4.88 | (-11.64, 2.39) | |
| All data (07/06 to 06/07) | Female | 2 | -1.48 | (-5.74, 2.98) | | -2.77 | (-9.21, 4.11) | | -4.58 | (-11.27, 2.63) | |
| All data (07/06 to 06/07) | Female | 0-2 | -0.78 | (-4.92, 3.54) | | -1.70 | (-8.30, 5.37) | | -5.08 | (-11.98, 2.36) | |
| All data (07/06 to 06/07) | Female | 0-3 | -1.08 | (-5.37, 3.39) | | -2.38 | (-9.16, 4.92) | | -5.55 | (-12.64, 2.11) | |
| All data (07/06 to 06/07) | Female | 0-4 | -1.01 | (-5.37, 3.56) | | -2.08 | (-9.03, 5.40) | | -6.25 | (-13.62, 1.75) | |
| All data (07/06 to 06/07) | Female | 0-8 | 0.23 | (-4.46, 5.14) | | 0.05 | (-7.54, 8.25) | | -7.17 | (-15.26, 1.69) | |
| All data (07/06 to 06/07) | Female | 0-12 | 0.24 | (-4.59, 5.31) | | 0.27 | (-7.52, 8.73) | | -5.75 | (-13.82, 3.07) | |
| All data (07/06 to 06/07) | Female | 0-24 | -0.43 | (-6.04, 5.50) | | -0.92 | (-9.63, 8.63) | | -5.98 | (-15.09, 4.10) | |
| All data (07/06 to 06/07) | Female | 0-48 | -1.84 | (-8.61, 5.43) | | -3.41 | (-13.35, 7.66) | | -1.42 | (-12.15, 10.63) | |
| All data (07/06 to 06/07) | Males, 35-64 years | 0 | 1.64 | (-2.79, 6.27) | | 0.01 | (-7.37, 7.99) | | 1.36 | (-7.31, 10.85) | |
| All data (07/06 to 06/07) | Males, 35-64 years | 1 | 1.83 | (-2.55, 6.41) | | 2.35 | (-4.99, 10.26) | | 1.99 | (-7.43, 12.38) | |
| All data (07/06 to 06/07) | Males, 35-64 years | 2 | 0.02 | (-4.37, 4.60) | | -0.62 | (-8.01, 7.36) | | -1.20 | (-10.57, 9.15) | |
| All data (07/06 to 06/07) | Males, 35-64 years | 0-2 | 1.80 | (-2.69, 6.50) | | 1.01 | (-6.57, 9.20) | | 2.65 | (-6.57, 12.79) | |
| All data (07/06 to 06/07) | Males, 35-64 years | 0-3 | 1.31 | (-3.23, 6.07) | | 0.12 | (-7.61, 8.50) | | 2.02 | (-7.64, 12.69) | |
| All data (07/06 to 06/07) | Males, 35-64 years | 0-4 | 0.96 | (-3.70, 5.84) | | 0.28 | (-7.63, 8.88) | | 2.50 | (-7.72, 13.86) | |
| All data (07/06 to 06/07) | Males, 35-64 years | 0-8 | 0.66 | (-4.50, 6.10) | | 0.68 | (-7.98, 10.15) | | 7.65 | (-4.53, 21.40) | |
| All data (07/06 to 06/07) | Males, 35-64 years | 0-12 | 1.59 | (-3.89, 7.38) | | 2.13 | (-7.08, 12.26) | | 8.81 | (-3.61, 22.83) | |
| All data (07/06 to 06/07) | Males, 35-64 years | 0-24 | 5.92 | (-0.77, 13.07) | * | 8.19 | (-2.72, 20.33) | | 17.16 | (2.72, 33.63) | ** |
| All data (07/06 to 06/07) | Males, 35-64 years | 0-48 | 9.35 | (0.63, 18.83) | ** | 11.11 | (-2.64, 26.81) | | 21.13 | (4.08, 40.97) | ** |
| All data (07/06 to 06/07) | Males, 65-74 years | 0 | 3.79 | (-3.81, 12.00) | | 1.56 | (-10.23, 14.90) | | -0.94 | (-13.08, 12.91) | |
| All data (07/06 to 06/07) | Males, 65-74 years | 1 | 3.34 | (-4.60, 11.93) | | 2.07 | (-10.04, 15.82) | | -1.57 | (-13.49, 11.98) | |
| All data (07/06 to 06/07) | Males, 65-74 years | 2 | 2.54 | (-5.37, 11.11) | | 7.49 | (-5.05, 21.69) | | 5.49 | (-6.97, 19.64) | |
| All data (07/06 to 06/07) | Males, 65-74 years | 0-2 | 3.58 | (-4.54, 12.38) | | 2.06 | (-10.53, 16.41) | | 1.00 | (-11.35, 15.07) | |

| Period[a] | Stratification | Lag (hours) | $PM_{2.5}$ % difference (95% CI) | | | $PM_{10}$ % difference (95% CI) | | CO % % difference (95% CI) | |
|---|---|---|---|---|---|---|---|---|---|
| All data (07/06 to 06/07) | Males, 65-74 years | 0-3 | 3.43 | (-4.95, 12.55) | | 4.33 | (-8.87, 19.44) | 1.60 | (-11.23, 16.28) |
| All data (07/06 to 06/07) | Males, 65-74 years | 0-4 | 3.27 | (-5.26, 12.57) | | 5.52 | (-8.11, 21.17) | 1.48 | (-11.47, 16.32) |
| All data (07/06 to 06/07) | Males, 65-74 years | 0-8 | 3.42 | (-5.21, 12.84) | | 7.16 | (-7.33, 23.91) | 1.40 | (-12.18, 17.08) |
| All data (07/06 to 06/07) | Males, 65-74 years | 0-12 | 2.57 | (-6.20, 12.15) | | 4.98 | (-9.27, 21.47) | -3.18 | (-16.29, 11.99) |
| All data (07/06 to 06/07) | Males, 65-74 years | 0-24 | 3.64 | (-6.35, 14.70) | | 3.84 | (-11.30, 21.57) | -1.71 | (-16.86, 16.19) |
| All data (07/06 to 06/07) | Males, 65-74 years | 0-48 | 3.46 | (-8.74, 17.28) | | 1.68 | (-15.93, 22.99) | 1.86 | (-16.29, 23.93) |
| All data (07/06 to 06/07) | Males, 75+ years | 0 | 2.23 | (-2.35, 7.03) | | 2.57 | (-4.86, 10.59) | 1.51 | (-6.68, 10.41) |
| All data (07/06 to 06/07) | Males, 75+ years | 1 | 3.04 | (-1.84, 8.17) | | 2.69 | (-5.05, 11.07) | 2.76 | (-5.39, 11.62) |
| All data (07/06 to 06/07) | Males, 75+ years | 2 | 1.34 | (-3.66, 6.61) | | 0.14 | (-7.64, 8.57) | 4.91 | (-3.30, 13.83) |
| All data (07/06 to 06/07) | Males, 75+ years | 0-2 | 2.66 | (-2.15, 7.70) | | 2.53 | (-5.31, 11.02) | 2.19 | (-6.04, 11.13) |
| All data (07/06 to 06/07) | Males, 75+ years | 0-3 | 2.28 | (-2.70, 7.52) | | 1.76 | (-6.33, 10.55) | 3.39 | (-5.12, 12.65) |
| All data (07/06 to 06/07) | Males, 75+ years | 0-4 | 2.63 | (-2.55, 8.08) | | 2.11 | (-6.29, 11.27) | 3.22 | (-5.54, 12.80) |
| All data (07/06 to 06/07) | Males, 75+ years | 0-8 | 4.10 | (-1.24, 9.72) | | 6.44 | (-2.62, 16.34) | 7.38 | (-2.78, 18.61) |
| All data (07/06 to 06/07) | Males, 75+ years | 0-12 | 3.88 | (-1.66, 9.73) | | 6.48 | (-2.89, 16.76) | 6.02 | (-4.11, 17.22) |
| All data (07/06 to 06/07) | Males, 75+ years | 0-24 | 4.40 | (-1.85, 11.05) | | 6.65 | (-3.45, 17.80) | 5.49 | (-5.93, 18.30) |
| All data (07/06 to 06/07) | Males, 75+ years | 0-48 | 8.13 | (0.52, 16.31) | ** | 9.34 | (-2.62, 22.76) | 5.90 | (-7.36, 21.06) |
| All data (07/06 to 06/07) | Females, 35-64 years | 0 | 0.54 | (-5.40, 6.86) | | 0.04 | (-9.91, 11.10) | -6.97 | (-20.68, 9.09) |
| All data (07/06 to 06/07) | Females, 35-64 years | 1 | -1.56 | (-8.10, 5.46) | | -3.69 | (-14.23, 8.15) | -10.31 | (-25.16, 7.50) |
| All data (07/06 to 06/07) | Females, 35-64 years | 2 | -1.54 | (-8.47, 5.93) | | -2.04 | (-13.02, 10.32) | -9.85 | (-25.35, 8.87) |
| All data (07/06 to 06/07) | Females, 35-64 years | 0-2 | -0.26 | (-6.54, 6.44) | | -1.44 | (-11.84, 10.19) | -8.36 | (-23.11, 9.22) |
| All data (07/06 to 06/07) | Females, 35-64 years | 0-3 | -0.74 | (-7.30, 6.29) | | -1.88 | (-12.67, 10.25) | -10.19 | (-25.59, 8.38) |
| All data (07/06 to 06/07) | Females, 35-64 years | 0-4 | -0.87 | (-7.61, 6.37) | | -1.92 | (-12.97, 10.54) | -12.59 | (-28.44, 6.77) |
| All data (07/06 to 06/07) | Females, 35-64 years | 0-8 | 0.25 | (-6.79, 7.81) | | -0.38 | (-12.25, 13.08) | -12.84 | (-30.56, 9.39) |
| All data (07/06 to 06/07) | Females, 35-64 years | 0-12 | 2.07 | (-5.63, 10.40) | | 1.78 | (-11.25, 16.72) | -6.06 | (-24.20, 16.42) |
| All data (07/06 to 06/07) | Females, 35-64 years | 0-24 | 3.82 | (-6.14, 14.83) | | 4.40 | (-11.95, 23.79) | -8.10 | (-27.83, 17.02) |
| All data (07/06 to 06/07) | Females, 35-64 years | 0-48 | 2.59 | (-11.47, 18.88) | | 2.48 | (-18.71, 29.20) | 4.93 | (-21.23, 39.80) |

| Period[a] | Stratification | Lag (hours) | $PM_{2.5}$ % difference (95% CI) | | $PM_{10}$ % difference (95% CI) | | CO % % difference (95% CI) | | |
|---|---|---|---|---|---|---|---|---|---|
| All data (07/06 to 06/07) | Females, 65-74 years | 0 | 5.05 | (-5.38, 16.64) | -0.40 | (-16.68, 19.05) | 9.83 | (-5.33, 27.41) | |
| All data (07/06 to 06/07) | Females, 65-74 years | 1 | 3.46 | (-6.51, 14.50) | 4.28 | (-11.69, 23.14) | 6.41 | (-8.74, 24.08) | |
| All data (07/06 to 06/07) | Females, 65-74 years | 2 | 3.38 | (-7.12, 15.07) | -0.22 | (-16.15, 18.74) | 5.16 | (-9.93, 22.79) | |
| All data (07/06 to 06/07) | Females, 65-74 years | 0-2 | 4.57 | (-5.76, 16.04) | 2.53 | (-13.99, 22.23) | 9.62 | (-6.09, 27.95) | |
| All data (07/06 to 06/07) | Females, 65-74 years | 0-3 | 4.41 | (-6.14, 16.14) | 1.33 | (-15.48, 21.49) | 8.67 | (-7.37, 27.50) | |
| All data (07/06 to 06/07) | Females, 65-74 years | 0-4 | 5.04 | (-5.78, 17.12) | 2.78 | (-14.80, 23.99) | 8.42 | (-8.48, 28.44) | |
| All data (07/06 to 06/07) | Females, 65-74 years | 0-8 | 7.96 | (-4.35, 21.87) | 4.21 | (-14.36, 26.81) | 7.50 | (-11.32, 30.32) | |
| All data (07/06 to 06/07) | Females, 65-74 years | 0-12 | 8.02 | (-4.29, 21.92) | 3.60 | (-14.68, 25.80) | 3.80 | (-14.44, 25.93) | |
| All data (07/06 to 06/07) | Females, 65-74 years | 0-24 | 5.87 | (-7.47, 21.14) | -1.95 | (-22.06, 23.35) | -7.03 | (-27.08, 18.54) | |
| All data (07/06 to 06/07) | Females, 65-74 years | 0-48 | 3.35 | (-13.09, 22.89) | -9.89 | (-32.12, 19.62) | -8.48 | (-30.27, 20.13) | |
| All data (07/06 to 06/07) | Females, 75+ years | 0 | -2.26 | (-8.51, 4.42) | -5.79 | (-14.96, 4.37) | -7.61 | (-16.27, 1.94) | |
| All data (07/06 to 06/07) | Females, 75+ years | 1 | -2.81 | (-9.31, 4.15) | 0.75 | (-8.68, 11.16) | -6.83 | (-15.31, 2.51) | |
| All data (07/06 to 06/07) | Females, 75+ years | 2 | -2.66 | (-8.94, 4.06) | -3.73 | (-12.56, 6.01) | -5.84 | (-14.14, 3.27) | |
| All data (07/06 to 06/07) | Females, 75+ years | 0-2 | -2.83 | (-9.36, 4.18) | -2.90 | (-12.56, 7.82) | -8.77 | (-17.43, 0.80) | * |
| All data (07/06 to 06/07) | Females, 75+ years | 0-3 | -3.03 | (-9.70, 4.13) | -3.69 | (-13.53, 7.26) | -8.65 | (-17.51, 1.17) | * |
| All data (07/06 to 06/07) | Females, 75+ years | 0-4 | -2.95 | (-9.71, 4.31) | -3.51 | (-13.54, 7.70) | -8.91 | (-18.10, 1.31) | * |
| All data (07/06 to 06/07) | Females, 75+ years | 0-8 | -2.63 | (-10.13, 5.50) | -1.06 | (-12.23, 11.53) | -10.20 | (-20.17, 1.01) | * |
| All data (07/06 to 06/07) | Females, 75+ years | 0-12 | -4.23 | (-11.70, 3.88) | -1.96 | (-12.99, 10.46) | -8.59 | (-18.58, 2.63) | |
| All data (07/06 to 06/07) | Females, 75+ years | 0-24 | -5.26 | (-13.15, 3.35) | -3.62 | (-15.08, 9.38) | -5.32 | (-16.66, 7.56) | |
| All data (07/06 to 06/07) | Females, 75+ years | 0-48 | -4.93 | (-13.50, 4.48) | -4.10 | (-16.46, 10.09) | -1.14 | (-14.32, 14.08) | |
| All data (07/06 to 06/07) | All data | 0 | 5.89 | (-4.60, 17.53) | -3.22 | (-10.86, 5.06) | -3.03 | (-7.12, 1.24) | |
| All data (07/06 to 06/07) | All data | 1 | 1.85 | (-8.09, 12.87) | 2.70 | (-5.48, 11.59) | -1.38 | (-5.50, 2.91) | |
| All data (07/06 to 06/07) | All data | 2 | 0.94 | (-8.54, 11.39) | 3.15 | (-5.24, 12.29) | -3.27 | (-7.45, 1.10) | |
| All data (07/06 to 06/07) | All data | 0-2 | 4.32 | (-6.08, 15.88) | 0.11 | (-8.00, 8.92) | -2.31 | (-6.49, 2.06) | |
| All data (07/06 to 06/07) | All data | 0-3 | 3.70 | (-6.76, 15.33) | 0.74 | (-7.77, 10.04) | -2.91 | (-7.27, 1.64) | |
| All data (07/06 to 06/07) | All data | 0-4 | 3.45 | (-7.04, 15.13) | 0.76 | (-8.11, 10.48) | -1.37 | (-5.95, 3.43) | |

| Period[a] | Stratification | Lag (hours) | $PM_{2.5}$ % difference (95% CI) | | | $PM_{10}$ % difference (95% CI) | | CO % % difference (95% CI) | |
|---|---|---|---|---|---|---|---|---|---|
| All data (07/06 to 06/07) | All data | 0-8 | 6.87 | (-4.19, 19.20) | | 2.47 | (-7.76, 13.83) | -1.94 | (-7.28, 3.71) |
| All data (07/06 to 06/07) | All data | 0-12 | 7.86 | (-4.07, 21.27) | | 1.13 | (-9.64, 13.17) | -2.75 | (-8.52, 3.39) |
| All data (07/06 to 06/07) | All data | 0-24 | 4.33 | (-8.67, 19.18) | | 1.76 | (-10.42, 15.59) | -2.60 | (-9.59, 4.94) |
| All data (07/06 to 06/07) | All data | 0-48 | -0.32 | (-14.23, 15.85) | | 7.26 | (-7.65, 24.57) | -0.56 | (-9.74, 9.54) |
| All data (07/06 to 06/07) | 35-64 years | 0 | -7.92 | (-23.16, 10.34) | | 2.06 | (-11.42, 17.59) | -1.10 | (-8.92, 7.39) |
| All data (07/06 to 06/07) | 35-64 years | 1 | -11.19 | (-25.92, 6.46) | | 4.85 | (-9.44, 21.39) | 0.01 | (-7.69, 8.35) |
| All data (07/06 to 06/07) | 35-64 years | 2 | -9.34 | (-23.93, 8.06) | | -0.27 | (-14.58, 16.44) | 2.27 | (-5.50, 10.67) |
| All data (07/06 to 06/07) | 35-64 years | 0-2 | -10.46 | (-25.58, 7.74) | | 5.10 | (-9.20, 21.66) | -0.54 | (-8.62, 8.26) |
| All data (07/06 to 06/07) | 35-64 years | 0-3 | -11.46 | (-26.76, 7.04) | | 4.14 | (-10.83, 21.61) | 0.62 | (-7.82, 9.84) |
| All data (07/06 to 06/07) | 35-64 years | 0-4 | -10.66 | (-26.23, 8.20) | | 1.10 | (-14.14, 19.06) | 1.76 | (-7.16, 11.54) |
| All data (07/06 to 06/07) | 35-64 years | 0-8 | -5.39 | (-22.34, 15.26) | | 1.12 | (-16.50, 22.45) | 4.24 | (-6.16, 15.79) |
| All data (07/06 to 06/07) | 35-64 years | 0-12 | -5.52 | (-23.63, 16.87) | | 3.89 | (-15.71, 28.06) | 5.23 | (-6.14, 17.99) |
| All data (07/06 to 06/07) | 35-64 years | 0-24 | -6.76 | (-26.87, 18.88) | | 15.07 | (-9.42, 46.19) | 6.11 | (-7.97, 22.34) |
| All data (07/06 to 06/07) | 35-64 years | 0-48 | -1.83 | (-25.02, 28.53) | | 20.86 | (-9.51, 61.44) | 2.27 | (-15.00, 23.05) |
| All data (07/06 to 06/07) | 65-74 years | 0 | 43.80 | (10.84, 86.54) | # | -5.77 | (-22.06, 13.94) | -4.78 | (-14.12, 5.56) |
| All data (07/06 to 06/07) | 65-74 years | 1 | 33.72 | (4.12, 71.74) | ** | 0.89 | (-16.50, 21.91) | -3.07 | (-12.46, 7.33) |
| All data (07/06 to 06/07) | 65-74 years | 2 | 23.49 | (-2.46, 56.35) | * | 3.91 | (-13.71, 25.13) | -2.53 | (-11.47, 7.31) |
| All data (07/06 to 06/07) | 65-74 years | 0-2 | 42.81 | (10.36, 84.81) | # | -2.31 | (-19.51, 18.57) | -4.15 | (-13.99, 6.82) |
| All data (07/06 to 06/07) | 65-74 years | 0-3 | 39.62 | (7.90, 80.67) | ** | -1.05 | (-18.93, 20.78) | -4.41 | (-14.41, 6.77) |
| All data (07/06 to 06/07) | 65-74 years | 0-4 | 36.10 | (5.25, 75.99) | ** | 1.38 | (-17.41, 24.46) | -0.86 | (-11.27, 10.76) |
| All data (07/06 to 06/07) | 65-74 years | 0-8 | 25.21 | (-3.60, 62.63) | * | 6.80 | (-14.64, 33.62) | -5.96 | (-17.88, 7.69) |
| All data (07/06 to 06/07) | 65-74 years | 0-12 | 19.89 | (-8.52, 57.13) | | 3.27 | (-18.71, 31.19) | -9.17 | (-21.72, 5.38) |
| All data (07/06 to 06/07) | 65-74 years | 0-24 | 23.70 | (-9.11, 68.37) | | -15.77 | (-36.99, 12.59) | -13.47 | (-27.44, 3.18) |
| All data (07/06 to 06/07) | 65-74 years | 0-48 | 2.41 | (-28.25, 46.18) | | -14.66 | (-39.09, 19.57) | -12.53 | (-29.79, 8.97) |
| All data (07/06 to 06/07) | 75+ years | 0 | 7.48 | (-7.62, 25.04) | | -7.19 | (-17.67, 4.62) | -3.62 | (-9.12, 2.23) |
| All data (07/06 to 06/07) | 75+ years | 1 | 2.99 | (-11.22, 19.48) | | 1.17 | (-10.28, 14.08) | -1.73 | (-7.32, 4.21) |

| Period[a] | Stratification | Lag (hours) | $PM_{2.5}$ % difference (95% CI) | | $PM_{10}$ % difference (95% CI) | | CO % % difference (95% CI) | | |
|---|---|---|---|---|---|---|---|---|---|
| All data (07/06 to 06/07) | 75+ years | 2 | 1.87 | (-11.54, 17.31) | 4.79 | (-7.28, 18.43) | -7.03 | (-12.87, , 0.79) | ** |
| All data (07/06 to 06/07) | 75+ years | 0-2 | 5.63 | (-9.29, 23.00) | -3.44 | (-14.62, 9.21) | -2.78 | (-8.33, 3.10) | |
| All data (07/06 to 06/07) | 75+ years | 0-3 | 5.37 | (-9.59, 22.80) | -1.71 | (-13.54, 11.75) | -4.29 | (-10.14, 1.94) | |
| All data (07/06 to 06/07) | 75+ years | 0-4 | 4.87 | (-10.02, 22.23) | -0.72 | (-13.17, 13.52) | -3.18 | (-9.32, 3.38) | |
| All data (07/06 to 06/07) | 75+ years | 0-8 | 10.48 | (-5.23, 28.79) | 0.11 | (-14.13, 16.72) | -4.05 | (-11.15, 3.60) | |
| All data (07/06 to 06/07) | 75+ years | 0-12 | 13.89 | (-3.50, 34.42) | -2.72 | (-17.29, 14.43) | -5.16 | (-12.79, 3.14) | |
| All data (07/06 to 06/07) | 75+ years | 0-24 | 7.25 | (-11.04, 29.30) | -0.44 | (-16.68, 18.96) | -4.27 | (-13.56, 6.02) | |
| All data (07/06 to 06/07) | 75+ years | 0-48 | 0.59 | (-18.59, 24.30) | 7.75 | (-12.29, 32.38) | 1.28 | (-11.42, 15.80) | |
| All data (07/06 to 06/07) | Male | 0 | 6.45 | (-6.59, 21.30) | -0.98 | (-10.67, 9.75) | -3.35 | (-8.26, 1.82) | |
| All data (07/06 to 06/07) | Male | 1 | 1.66 | (-10.68, 15.69) | 4.90 | (-5.51, 16.45) | -1.13 | (-6.16, 4.17) | |
| All data (07/06 to 06/07) | Male | 2 | 1.13 | (-10.56, 14.36) | 5.19 | (-5.51, 17.09) | -2.27 | (-7.50, 3.26) | |
| All data (07/06 to 06/07) | Male | 0-2 | 4.44 | (-8.50, 19.21) | 3.21 | (-7.16, 14.73) | -2.16 | (-7.19, 3.15) | |
| All data (07/06 to 06/07) | Male | 0-3 | 3.68 | (-9.28, 18.49) | 3.92 | (-7.00, 16.12) | -2.20 | (-7.47, 3.38) | |
| All data (07/06 to 06/07) | Male | 0-4 | 3.87 | (-9.14, 18.75) | 3.14 | (-8.13, 15.79) | -1.73 | (-7.34, 4.23) | |
| All data (07/06 to 06/07) | Male | 0-8 | 3.81 | (-9.41, 18.95) | 7.78 | (-5.45, 22.87) | -1.13 | (-7.84, 6.07) | |
| All data (07/06 to 06/07) | Male | 0-12 | 3.62 | (-10.48, 19.95) | 7.15 | (-6.84, 23.23) | -1.31 | (-8.65, 6.61) | |
| All data (07/06 to 06/07) | Male | 0-24 | 1.77 | (-13.82, 20.18) | 8.63 | (-7.39, 27.42) | -0.71 | (-9.58, 9.03) | |
| All data (07/06 to 06/07) | Male | 0-48 | 0.70 | (-16.53, 21.47) | 13.17 | (-6.35, 36.76) | 4.45 | (-7.54, 18.00) | |
| All data (07/06 to 06/07) | Female | 0 | 6.76 | (-10.25, 27.00) | -8.03 | (-19.84, 5.51) | -2.73 | (-9.95, 5.07) | |
| All data (07/06 to 06/07) | Female | 1 | 3.49 | (-12.60, 22.54) | -1.66 | (-14.31, 12.85) | -2.18 | (-9.15, 5.33) | |
| All data (07/06 to 06/07) | Female | 2 | 1.67 | (-13.82, 19.94) | -0.82 | (-13.74, 14.04) | -5.60 | (-12.48, 1.82) | |
| All data (07/06 to 06/07) | Female | 0-2 | 5.70 | (-11.11, 25.69) | -5.97 | (-18.36, 8.29) | -3.01 | (-10.30, 4.88) | |
| All data (07/06 to 06/07) | Female | 0-3 | 5.23 | (-11.75, 25.47) | -5.38 | (-18.30, 9.59) | -4.88 | (-12.30, 3.18) | |
| All data (07/06 to 06/07) | Female | 0-4 | 4.17 | (-12.81, 24.46) | -4.24 | (-17.86, 11.64) | -1.22 | (-8.91, 7.13) | |
| All data (07/06 to 06/07) | Female | 0-8 | 13.92 | (-5.15, 36.82) | -7.42 | (-22.48, 10.57) | -3.92 | (-12.45, 5.46) | |
| All data (07/06 to 06/07) | Female | 0-12 | 17.05 | (-3.82, 42.44) | -9.90 | (-25.52, 9.00) | -5.58 | (-14.62, 4.43) | |

| Period[a] | Stratification | Lag (hours) | $PM_{2.5}$ % difference (95% CI) | | | $PM_{10}$ % difference (95% CI) | | CO % % difference (95% CI) | | |
|---|---|---|---|---|---|---|---|---|---|---|
| All data (07/06 to 06/07) | Female | 0-24 | 9.56 | (-12.28, 36.85) | | -10.09 | (-27.29, 11.17) | -6.12 | (-17.05, 6.26) | |
| All data (07/06 to 06/07) | Female | 0-48 | -2.49 | (-24.20, 25.44) | | -2.76 | (-23.92, 24.30) | -8.88 | (-22.36, 6.95) | |
| All data (07/06 to 06/07) | Males, 35-64 years | 0 | -12.21 | (-29.19, 8.83) | | 1.60 | (-14.17, 20.27) | -1.65 | (-10.44, 8.01) | |
| All data (07/06 to 06/07) | Males, 35-64 years | 1 | -15.88 | (-32.34, 4.58) | | 4.23 | (-12.41, 24.04) | -0.63 | (-9.37, 8.95) | |
| All data (07/06 to 06/07) | Males, 35-64 years | 2 | -11.42 | (-27.92, 8.86) | | 0.25 | (-16.37, 20.18) | 2.80 | (-6.10, 12.54) | |
| All data (07/06 to 06/07) | Males, 35-64 years | 0-2 | -15.10 | (-31.94, 5.91) | | 4.44 | (-12.22, 24.26) | -1.05 | (-10.24, 9.07) | |
| All data (07/06 to 06/07) | Males, 35-64 years | 0-3 | -16.00 | (-33.03, 5.36) | | 4.21 | (-13.30, 25.26) | 0.74 | (-8.92, 11.42) | |
| All data (07/06 to 06/07) | Males, 35-64 years | 0-4 | -15.51 | (-32.77, 6.17) | | 1.76 | (-16.06, 23.36) | 2.09 | (-8.24, 13.58) | |
| All data (07/06 to 06/07) | Males, 35-64 years | 0-8 | -14.40 | (-32.52, 8.58) | | 8.08 | (-13.61, 35.22) | 8.09 | (-4.41, 22.23) | |
| All data (07/06 to 06/07) | Males, 35-64 years | 0-12 | -16.86 | (-35.53, 7.23) | | 12.29 | (-11.94, 43.19) | 10.22 | (-3.54, 25.95) | |
| All data (07/06 to 06/07) | Males, 35-64 years | 0-24 | -14.48 | (-35.81, 13.93) | | 24.54 | (-5.43, 63.99) | 12.98 | (-4.25, 33.31) | |
| All data (07/06 to 06/07) | Males, 35-64 years | 0-48 | -4.80 | (-30.50, 30.41) | | 26.96 | (-8.91, 76.98) | 8.26 | (-12.56, 34.04) | |
| All data (07/06 to 06/07) | Males, 65-74 years | 0 | 41.86 | (1.91, 97.49) | ** | -8.40 | (-28.02, 16.57) | -5.84 | (-16.78, 6.53) | |
| All data (07/06 to 06/07) | Males, 65-74 years | 1 | 28.93 | (-5.92, 76.70) | | -0.42 | (-21.34, 26.05) | -2.41 | (-13.55, 10.16) | |
| All data (07/06 to 06/07) | Males, 65-74 years | 2 | 24.87 | (-7.09, 67.81) | | 2.20 | (-19.07, 29.07) | -1.98 | (-13.42, 10.97) | |
| All data (07/06 to 06/07) | Males, 65-74 years | 0-2 | 40.03 | (1.09, 93.97) | ** | -3.59 | (-24.38, 22.91) | -4.74 | (-16.37, 8.50) | |
| All data (07/06 to 06/07) | Males, 65-74 years | 0-3 | 38.37 | (-0.17, 91.78) | * | -2.84 | (-24.40, 24.88) | -4.36 | (-16.53, 9.59) | |
| All data (07/06 to 06/07) | Males, 65-74 years | 0-4 | 36.67 | (-1.16, 88.98) | * | -0.91 | (-23.45, 28.27) | -5.34 | (-18.08, 9.38) | |
| All data (07/06 to 06/07) | Males, 65-74 years | 0-8 | 27.93 | (-7.28, 76.50) | | 3.74 | (-21.36, 36.86) | -12.09 | (-25.77, 4.11) | |
| All data (07/06 to 06/07) | Males, 65-74 years | 0-12 | 25.03 | (-10.18, 74.06) | | 0.65 | (-24.75, 34.62) | -14.82 | (-29.15, 2.39) | * |
| All data (07/06 to 06/07) | Males, 65-74 years | 0-24 | 27.19 | (-13.15, 86.26) | | -13.83 | (-39.65, 23.04) | -15.82 | (-32.00, 4.22) | |
| All data (07/06 to 06/07) | Males, 65-74 years | 0-48 | 10.00 | (-29.28, 71.10) | | -13.69 | (-43.04, 30.79) | -10.17 | (-30.99, 16.94) | |
| All data (07/06 to 06/07) | Males, 75+ years | 0 | 15.44 | (-5.27, 40.68) | | -1.85 | (-16.00, 14.67) | -3.55 | (-10.43, 3.86) | |
| All data (07/06 to 06/07) | Males, 75+ years | 1 | 9.68 | (-9.69, 33.21) | | 6.49 | (-9.15, 24.82) | -1.08 | (-8.26, 6.67) | |
| All data (07/06 to 06/07) | Males, 75+ years | 2 | 4.69 | (-12.88, 25.81) | | 10.31 | (-6.38, 29.98) | -6.03 | (-13.72, 2.34) | |
| All data (07/06 to 06/07) | Males, 75+ years | 0-2 | 12.88 | (-7.55, 37.82) | | 3.47 | (-11.93, 21.56) | -1.99 | (-8.85, 5.39) | |

| Period[a] | Stratification | Lag (hours) | $PM_{2.5}$ % difference (95% CI) | | | $PM_{10}$ % difference (95% CI) | | CO % % difference (95% CI) | |
|---|---|---|---|---|---|---|---|---|---|
| All data (07/06 to 06/07) | Males, 75+ years | 0-3 | 11.65 | (-8.52, 36.27) | | 5.34 | (-11.01, 24.69) | -3.00 | (-10.28, 4.87) |
| All data (07/06 to 06/07) | Males, 75+ years | 0-4 | 11.53 | (-8.56, 36.02) | | 4.74 | (-12.22, 24.97) | -2.55 | (-10.30, 5.87) |
| All data (07/06 to 06/07) | Males, 75+ years | 0-8 | 11.40 | (-8.55, 35.70) | | 7.65 | (-11.93, 31.57) | -2.71 | (-12.09, 7.66) |
| All data (07/06 to 06/07) | Males, 75+ years | 0-12 | 13.76 | (-8.26, 41.07) | | 4.89 | (-15.30, 29.89) | -3.81 | (-14.03, 7.61) |
| All data (07/06 to 06/07) | Males, 75+ years | 0-24 | 7.93 | (-15.39, 37.69) | | 5.62 | (-16.73, 33.97) | -4.02 | (-16.26, 10.02) |
| All data (07/06 to 06/07) | Males, 75+ years | 0-48 | 2.20 | (-22.53, 34.83) | | 16.17 | (-12.08, 53.50) | 7.70 | (-10.23, 29.22) |
| All data (07/06 to 06/07) | Females, 35-64 years | 0 | 5.81 | (-24.11, 47.52) | | 2.76 | (-20.92, 33.52) | 0.45 | (-15.56, 19.49) |
| All data (07/06 to 06/07) | Females, 35-64 years | 1 | 4.71 | (-23.99, 44.26) | | 4.48 | (-20.60, 37.48) | 1.66 | (-13.73, 19.79) |
| All data (07/06 to 06/07) | Females, 35-64 years | 2 | -0.77 | (-28.96, 38.59) | | -1.98 | (-27.48, 32.47) | 0.62 | (-14.48, 18.38) |
| All data (07/06 to 06/07) | Females, 35-64 years | 0-2 | 4.98 | (-24.61, 46.18) | | 5.71 | (-19.51, 38.84) | 0.83 | (-15.15, 19.81) |
| All data (07/06 to 06/07) | Females, 35-64 years | 0-3 | 3.83 | (-26.15, 45.99) | | 3.24 | (-22.79, 38.04) | 0.15 | (-16.19, 19.66) |
| All data (07/06 to 06/07) | Females, 35-64 years | 0-4 | 5.88 | (-25.11, 49.68) | | -1.25 | (-27.72, 34.92) | 0.77 | (-15.94, 20.79) |
| All data (07/06 to 06/07) | Females, 35-64 years | 0-8 | 23.58 | (-13.47, 76.50) | | -15.43 | (-41.95, 23.20) | -5.56 | (-23.15, 16.07) |
| All data (07/06 to 06/07) | Females, 35-64 years | 0-12 | 33.94 | (-9.47, 98.16) | | -16.98 | (-45.33, 26.07) | -8.21 | (-26.74, 14.99) |
| All data (07/06 to 06/07) | Females, 35-64 years | 0-24 | 22.07 | (-23.00, 93.54) | | -12.11 | (-46.07, 43.23) | -12.70 | (-34.23, 15.87) |
| All data (07/06 to 06/07) | Females, 35-64 years | 0-48 | 13.56 | (-33.25, 93.19) | | 0.70 | (-44.25, 81.86) | -15.13 | (-41.65, 23.46) |
| All data (07/06 to 06/07) | Females, 65-74 years | 0 | 49.33 | (-2.37, 128.42) | * | -2.27 | (-28.39, 33.38) | -2.12 | (-19.03, 18.34) |
| All data (07/06 to 06/07) | Females, 65-74 years | 1 | 43.48 | (-5.33, 117.44) | * | 3.34 | (-24.90, 42.20) | -4.26 | (-20.57, 15.40) |
| All data (07/06 to 06/07) | Females, 65-74 years | 2 | 22.97 | (-17.19, 82.60) | | 6.17 | (-22.01, 44.52) | -3.94 | (-17.91, 12.41) |
| All data (07/06 to 06/07) | Females, 65-74 years | 0-2 | 49.32 | (-2.38, 128.41) | * | -0.72 | (-28.16, 37.20) | -2.57 | (-19.92, 18.54) |
| All data (07/06 to 06/07) | Females, 65-74 years | 0-3 | 43.30 | (-6.13, 118.76) | * | 1.38 | (-27.09, 40.96) | -4.65 | (-21.13, 15.27) |
| All data (07/06 to 06/07) | Females, 65-74 years | 0-4 | 36.62 | (-10.61, 108.83) | | 4.54 | (-25.50, 46.69) | 5.95 | (-10.82, 25.87) |
| All data (07/06 to 06/07) | Females, 65-74 years | 0-8 | 20.56 | (-23.22, 89.32) | | 11.84 | (-23.65, 63.83) | 6.66 | (-15.36, 34.41) |
| All data (07/06 to 06/07) | Females, 65-74 years | 0-12 | 10.05 | (-31.55, 76.91) | | 8.08 | (-29.13, 64.84) | 2.64 | (-20.45, 32.42) |
| All data (07/06 to 06/07) | Females, 65-74 years | 0-24 | 15.16 | (-31.98, 94.97) | | -18.92 | (-50.79, 33.60) | -7.25 | (-32.20, 26.89) |
| All data (07/06 to 06/07) | Females, 65-74 years | 0-48 | -13.87 | (-53.07, 58.09) | | -13.95 | (-51.75, 53.45) | -16.24 | (-43.86, 24.96) |

| Period[a] | Stratification | Lag (hours) | $PM_{2.5}$ % difference (95% CI) | | | $PM_{10}$ % difference (95% CI) | | | CO % % difference (95% CI) | | |
|---|---|---|---|---|---|---|---|---|---|---|---|
| All data (07/06 to 06/07) | Females, 75+ years | 0 | -2.82 | (-23.30, 23.13) | | -14.56 | (-29.18, 3.08) | | -3.70 | (-12.63, 6.14) | |
| All data (07/06 to 06/07) | Females, 75+ years | 1 | -5.68 | (-25.13, 18.82) | | -5.64 | (-21.46, 13.36) | | -2.73 | (-11.38, 6.77) | |
| All data (07/06 to 06/07) | Females, 75+ years | 2 | -2.02 | (-21.39, 22.14) | | -2.00 | (-18.47, 17.79) | | -8.27 | (-17.03, 1.40) | * |
| All data (07/06 to 06/07) | Females, 75+ years | 0-2 | -3.64 | (-23.93, 22.06) | | -12.41 | (-27.63, 6.01) | | -4.18 | (-13.20, 5.78) | |
| All data (07/06 to 06/07) | Females, 75+ years | 0-3 | -2.96 | (-23.64, 23.33) | | -10.76 | (-26.76, 8.73) | | -6.45 | (-15.76, 3.90) | |
| All data (07/06 to 06/07) | Females, 75+ years | 0-4 | -4.04 | (-24.60, 22.11) | | -7.90 | (-25.03, 13.15) | | -4.16 | (-13.87, 6.64) | |
| All data (07/06 to 06/07) | Females, 75+ years | 0-8 | 9.16 | (-14.48, 39.32) | | -9.76 | (-28.90, 14.53) | | -5.85 | (-16.32, 5.93) | |
| All data (07/06 to 06/07) | Females, 75+ years | 0-12 | 13.99 | (-12.15, 47.89) | | -12.20 | (-31.64, 12.75) | | -6.83 | (-17.89, 5.73) | |
| All data (07/06 to 06/07) | Females, 75+ years | 0-24 | 6.37 | (-20.59, 42.48) | | -7.79 | (-29.54, 20.68) | | -4.66 | (-18.28, 11.23) | |
| All data (07/06 to 06/07) | Females, 75+ years | 0-48 | -1.49 | (-29.03, 36.74) | | -1.19 | (-27.27, 34.24) | | -5.51 | (-22.52, 15.23) | |
| Fire Season, (11/06 to 3/07) | All data | 0 | 1.93 | (-0.57, 4.50) | | 2.96 | (-1.41, 7.53) | | 3.89 | (-5.96, 14.78) | |
| Fire Season, (11/06 to 3/07) | All data | 1 | 1.49 | (-1.11, 4.15) | | 3.36 | (-1.12, 8.04) | | 0.11 | (-10.09, 11.48) | |
| Fire Season, (11/06 to 3/07) | All data | 2 | 0.38 | (-2.27, 3.10) | | 0.91 | (-3.55, 5.57) | | 0.23 | (-10.30, 12.00) | |
| Fire Season, (11/06 to 3/07) | All data | 0-2 | 1.69 | (-0.91, 4.36) | | 3.20 | (-1.36, 7.97) | | 3.58 | (-6.75, 15.05) | |
| Fire Season, (11/06 to 3/07) | All data | 0-3 | 1.35 | (-1.33, 4.09) | | 2.48 | (-2.20, 7.37) | | 2.41 | (-8.34, 14.41) | |
| Fire Season, (11/06 to 3/07) | All data | 0-4 | 1.30 | (-1.45, 4.12) | | 2.77 | (-2.04, 7.81) | | 1.77 | (-9.37, 14.28) | |
| Fire Season, (11/06 to 3/07) | All data | 0-8 | 1.99 | (-0.98, 5.05) | | 4.46 | (-0.79, 9.99) | * | 5.03 | (-7.72, 19.54) | |
| Fire Season, (11/06 to 3/07) | All data | 0-12 | 2.32 | (-0.78, 5.53) | | 5.39 | (-0.08, 11.16) | * | 8.91 | (-4.83, 24.64) | |
| Fire Season, (11/06 to 3/07) | All data | 0-24 | 3.54 | (-0.09, 7.31) | * | 6.99 | (0.80, 13.56) | ** | 16.49 | (-0.10, 35.83) | * |
| Fire Season, (11/06 to 3/07) | All data | 0-48 | 5.42 | (0.87, 10.17) | ** | 7.75 | (0.28, 15.77) | ** | 24.36 | (4.46, 48.04) | ** |
| Fire Season, (11/06 to 3/07) | 35-64 years | 0 | 1.62 | (-2.11, 5.48) | | 1.86 | (-4.65, 8.82) | | 4.34 | (-10.82, 22.07) | |
| Fire Season, (11/06 to 3/07) | 35-64 years | 1 | 0.55 | (-3.25, 4.50) | | 1.02 | (-5.50, 7.99) | | -1.26 | (-16.72, 17.07) | |
| Fire Season, (11/06 to 3/07) | 35-64 years | 2 | -1.00 | (-4.98, 3.14) | | -1.51 | (-8.30, 5.77) | | -6.72 | (-22.44, 12.19) | |
| Fire Season, (11/06 to 3/07) | 35-64 years | 0-2 | 1.14 | (-2.68, 5.11) | | 1.55 | (-5.12, 8.70) | | 3.90 | (-11.89, 22.50) | |
| Fire Season, (11/06 to 3/07) | 35-64 years | 0-3 | 0.52 | (-3.40, 4.60) | | 0.31 | (-6.53, 7.66) | | 0.94 | (-15.39, 20.41) | |
| Fire Season, (11/06 to 3/07) | 35-64 years | 0-4 | 0.14 | (-3.90, 4.36) | | -0.17 | (-7.18, 7.37) | | -1.68 | (-18.44, 18.53) | |

| Period[a] | Stratification | Lag (hours) | $PM_{2.5}$ % difference (95% CI) | | | $PM_{10}$ % difference (95% CI) | | | CO % % difference (95% CI) | | |
|---|---|---|---|---|---|---|---|---|---|---|---|
| Fire Season, (11/06 to 3/07) | 35-64 years | 0-8 | -0.03 | (-4.46, 4.60) | | -0.37 | (-7.96, 7.85) | | -4.21 | (-22.64, 18.61) | |
| Fire Season, (11/06 to 3/07) | 35-64 years | 0-12 | 1.29 | (-3.46, 6.28) | | 1.72 | (-6.48, 10.63) | | 4.30 | (-16.19, 29.80) | |
| Fire Season, (11/06 to 3/07) | 35-64 years | 0-24 | 5.31 | (-0.64, 11.62) | * | 8.44 | (-1.71, 19.64) | | 27.74 | (-1.42, 65.52) | * |
| Fire Season, (11/06 to 3/07) | 35-64 years | 0-48 | 7.74 | (-0.32, 16.46) | * | 9.47 | (-3.63, 24.35) | | 42.55 | (3.97, 95.44) | ** |
| Fire Season, (11/06 to 3/07) | 65-74 years | 0 | 4.69 | (-2.12, 11.97) | | 7.21 | (-4.36, 20.19) | | 8.13 | (-15.94, 39.10) | |
| Fire Season, (11/06 to 3/07) | 65-74 years | 1 | 3.02 | (-3.77, 10.30) | | 5.60 | (-6.02, 18.66) | | -5.00 | (-27.29, 24.13) | |
| Fire Season, (11/06 to 3/07) | 65-74 years | 2 | 3.04 | (-3.91, 10.50) | | 6.02 | (-5.63, 19.11) | | -1.65 | (-24.84, 28.69) | |
| Fire Season, (11/06 to 3/07) | 65-74 years | 0-2 | 3.90 | (-3.12, 11.43) | | 6.85 | (-5.26, 20.52) | | 6.16 | (-18.10, 37.61) | |
| Fire Season, (11/06 to 3/07) | 65-74 years | 0-3 | 3.80 | (-3.42, 11.56) | | 7.21 | (-5.33, 21.41) | | 3.21 | (-21.39, 35.51) | |
| Fire Season, (11/06 to 3/07) | 65-74 years | 0-4 | 3.97 | (-3.42, 11.93) | | 8.48 | (-4.59, 23.35) | | 3.94 | (-21.09, 36.93) | |
| Fire Season, (11/06 to 3/07) | 65-74 years | 0-8 | 5.70 | (-2.14, 14.17) | | 11.45 | (-2.87, 27.87) | | 12.77 | (-15.71, 50.88) | |
| Fire Season, (11/06 to 3/07) | 65-74 years | 0-12 | 5.78 | (-2.17, 14.39) | | 10.09 | (-3.85, 26.05) | | 17.74 | (-14.00, 61.20) | |
| Fire Season, (11/06 to 3/07) | 65-74 years | 0-24 | 6.24 | (-2.73, 16.04) | | 8.85 | (-5.91, 25.92) | | 18.95 | (-17.42, 71.34) | |
| Fire Season, (11/06 to 3/07) | 65-74 years | 0-48 | 6.10 | (-5.03, 18.53) | | 6.60 | (-10.71, 27.27) | | 19.49 | (-21.99, 83.03) | |
| Fire Season, (11/06 to 3/07) | 75+ years | 0 | 1.57 | (-2.32, 5.61) | | 3.09 | (-3.61, 10.24) | | 1.86 | (-12.52, 18.60) | |
| Fire Season, (11/06 to 3/07) | 75+ years | 1 | 2.03 | (-2.11, 6.34) | | 5.69 | (-1.42, 13.30) | | 3.28 | (-12.36, 21.70) | |
| Fire Season, (11/06 to 3/07) | 75+ years | 2 | 1.04 | (-3.16, 5.42) | | 1.95 | (-4.88, 9.27) | | 7.22 | (-9.15, 26.55) | |
| Fire Season, (11/06 to 3/07) | 75+ years | 0-2 | 1.69 | (-2.39, 5.93) | | 4.26 | (-2.84, 11.87) | | 2.15 | (-13.12, 20.09) | |
| Fire Season, (11/06 to 3/07) | 75+ years | 0-3 | 1.58 | (-2.65, 5.98) | | 3.77 | (-3.52, 11.61) | | 3.35 | (-12.75, 22.41) | |
| Fire Season, (11/06 to 3/07) | 75+ years | 0-4 | 1.82 | (-2.53, 6.36) | | 4.72 | (-2.82, 12.85) | | 3.91 | (-13.01, 24.12) | |
| Fire Season, (11/06 to 3/07) | 75+ years | 0-8 | 2.97 | (-1.69, 7.86) | | 7.99 | (-0.31, 16.98) | * | 9.07 | (-10.69, 33.21) | |
| Fire Season, (11/06 to 3/07) | 75+ years | 0-12 | 2.17 | (-2.60, 7.18) | | 7.89 | (-0.50, 17.00) | * | 8.50 | (-11.85, 33.54) | |
| Fire Season, (11/06 to 3/07) | 75+ years | 0-24 | 1.41 | (-3.88, 6.98) | | 5.97 | (-2.93, 15.69) | | 7.44 | (-14.36, 34.80) | |
| Fire Season, (11/06 to 3/07) | 75+ years | 0-48 | 4.11 | (-2.09, 10.71) | | 7.85 | (-2.40, 19.18) | | 16.45 | (-8.54, 48.27) | |
| Fire Season, (11/06 to 3/07) | Male | 0 | 2.48 | (-0.66, 5.72) | | 4.45 | (-1.05, 10.26) | | 6.91 | (-5.80, 21.34) | |
| Fire Season, (11/06 to 3/07) | Male | 1 | 2.33 | (-0.90, 5.67) | | 4.15 | (-1.43, 10.05) | | 4.66 | (-8.40, 19.59) | |

| Period[a] | Stratification | Lag (hours) | $PM_{2.5}$ % difference (95% CI) | | | $PM_{10}$ % difference (95% CI) | | | CO % % difference (95% CI) | | |
|---|---|---|---|---|---|---|---|---|---|---|---|
| Fire Season, (11/06 to 3/07) | Male | 2 | 0.58 | (-2.69, 3.95) | | 1.18 | (-4.37, 7.06) | | 4.37 | (-8.83, 19.47) | |
| Fire Season, (11/06 to 3/07) | Male | 0-2 | 2.38 | (-0.86, 5.74) | | 4.31 | (-1.41, 10.36) | | 7.70 | (-5.59, 22.86) | |
| Fire Season, (11/06 to 3/07) | Male | 0-3 | 1.92 | (-1.40, 5.36) | | 3.33 | (-2.50, 9.52) | | 7.59 | (-6.19, 23.40) | |
| Fire Season, (11/06 to 3/07) | Male | 0-4 | 1.79 | (-1.63, 5.34) | | 3.56 | (-2.46, 9.95) | | 6.61 | (-7.63, 23.04) | |
| Fire Season, (11/06 to 3/07) | Male | 0-8 | 1.97 | (-1.70, 5.77) | | 4.33 | (-2.15, 11.25) | | 12.04 | (-4.40, 31.30) | |
| Fire Season, (11/06 to 3/07) | Male | 0-12 | 2.48 | (-1.37, 6.49) | | 5.52 | (-1.28, 12.79) | | 16.30 | (-1.61, 37.48) | * |
| Fire Season, (11/06 to 3/07) | Male | 0-24 | 4.65 | (0.13, 9.39) | ** | 8.34 | (0.63, 16.64) | ** | 24.58 | (2.90, 50.81) | ** |
| Fire Season, (11/06 to 3/07) | Male | 0-48 | 8.05 | (2.30, 14.13) | # | 11.07 | (1.55, 21.48) | ** | 35.68 | (8.98, 68.92) | # |
| Fire Season, (11/06 to 3/07) | Female | 0 | 0.89 | (-3.26, 5.22) | | 0.38 | (-6.79, 8.09) | | -1.32 | (-16.18, 16.18) | |
| Fire Season, (11/06 to 3/07) | Female | 1 | -0.11 | (-4.49, 4.46) | | 1.97 | (-5.36, 9.86) | | -8.22 | (-23.81, 10.55) | |
| Fire Season, (11/06 to 3/07) | Female | 2 | -0.06 | (-4.58, 4.66) | | 0.61 | (-6.74, 8.54) | | -7.96 | (-24.52, 12.22) | |
| Fire Season, (11/06 to 3/07) | Female | 0-2 | 0.39 | (-3.94, 4.91) | | 1.27 | (-6.16, 9.30) | | -3.75 | (-19.39, 14.93) | |
| Fire Season, (11/06 to 3/07) | Female | 0-3 | 0.23 | (-4.24, 4.92) | | 1.02 | (-6.63, 9.29) | | -7.11 | (-23.42, 12.66) | |
| Fire Season, (11/06 to 3/07) | Female | 0-4 | 0.35 | (-4.21, 5.14) | | 1.48 | (-6.35, 9.96) | | -7.28 | (-24.22, 13.46) | |
| Fire Season, (11/06 to 3/07) | Female | 0-8 | 1.90 | (-3.09, 7.14) | | 4.72 | (-3.99, 14.23) | | -8.44 | (-27.19, 15.14) | |
| Fire Season, (11/06 to 3/07) | Female | 0-12 | 1.83 | (-3.33, 7.27) | | 5.09 | (-3.87, 14.88) | | -4.52 | (-24.35, 20.50) | |
| Fire Season, (11/06 to 3/07) | Female | 0-24 | 1.14 | (-4.92, 7.59) | | 4.08 | (-6.04, 15.29) | | 0.94 | (-22.45, 31.40) | |
| Fire Season, (11/06 to 3/07) | Female | 0-48 | 0.16 | (-7.25, 8.16) | | 1.26 | (-10.41, 14.44) | | 4.33 | (-22.13, 39.80) | |
| Fire Season, (11/06 to 3/07) | Males, 35-64 years | 0 | 2.13 | (-2.50, 6.99) | | 3.02 | (-5.11, 11.84) | | 11.99 | (-7.82, 36.05) | |
| Fire Season, (11/06 to 3/07) | Males, 35-64 years | 1 | 1.38 | (-3.23, 6.22) | | 3.27 | (-4.72, 11.94) | | 5.50 | (-14.00, 29.44) | |
| Fire Season, (11/06 to 3/07) | Males, 35-64 years | 2 | -0.78 | (-5.49, 4.16) | | -1.05 | (-9.17, 7.79) | | -3.85 | (-22.43, 19.17) | |
| Fire Season, (11/06 to 3/07) | Males, 35-64 years | 0-2 | 1.82 | (-2.88, 6.74) | | 3.34 | (-4.95, 12.34) | | 11.67 | (-8.69, 36.55) | |
| Fire Season, (11/06 to 3/07) | Males, 35-64 years | 0-3 | 1.10 | (-3.67, 6.11) | | 1.61 | (-6.79, 10.78) | | 7.85 | (-12.76, 33.32) | |
| Fire Season, (11/06 to 3/07) | Males, 35-64 years | 0-4 | 0.65 | (-4.26, 5.80) | | 0.97 | (-7.60, 10.35) | | 4.21 | (-16.74, 30.44) | |
| Fire Season, (11/06 to 3/07) | Males, 35-64 years | 0-8 | -0.12 | (-5.61, 5.67) | | -0.07 | (-9.41, 10.23) | | 1.41 | (-21.92, 31.72) | |
| Fire Season, (11/06 to 3/07) | Males, 35-64 years | 0-12 | 1.00 | (-4.82, 7.17) | | 2.03 | (-7.94, 13.07) | | 8.38 | (-17.08, 41.67) | |

| Period[a] | Stratification | Lag (hours) | $PM_{2.5}$ % difference (95% CI) | | | $PM_{10}$ % difference (95% CI) | | | CO % % difference (95% CI) | | |
|---|---|---|---|---|---|---|---|---|---|---|---|
| Fire Season, (11/06 to 3/07) | Males, 35-64 years | 0-24 | 5.85 | (-1.28, 13.49) | | 9.73 | (-2.38, 23.33) | | 33.60 | (-1.92, 81.99) | * |
| Fire Season, (11/06 to 3/07) | Males, 35-64 years | 0-48 | 9.48 | (0.11, 19.72) | ** | 12.51 | (-2.88, 30.33) | | 46.89 | (1.74, 112.07) | ** |
| Fire Season, (11/06 to 3/07) | Males, 65-74 years | 0 | 4.35 | (-4.10, 13.55) | | 9.81 | (-4.67, 26.48) | | -4.42 | (-31.86, 34.07) | |
| Fire Season, (11/06 to 3/07) | Males, 65-74 years | 1 | 2.59 | (-6.15, 12.14) | | 5.33 | (-9.34, 22.37) | | -11.39 | (-37.84, 26.31) | |
| Fire Season, (11/06 to 3/07) | Males, 65-74 years | 2 | 2.51 | (-6.21, 12.05) | | 8.25 | (-6.71, 25.60) | | -2.02 | (-30.37, 37.87) | |
| Fire Season, (11/06 to 3/07) | Males, 65-74 years | 0-2 | 3.20 | (-5.76, 13.01) | | 8.07 | (-7.31, 26.01) | | -4.31 | (-32.60, 35.86) | |
| Fire Season, (11/06 to 3/07) | Males, 65-74 years | 0-3 | 3.11 | (-6.14, 13.27) | | 9.18 | (-6.94, 28.08) | | -3.45 | (-32.73, 38.58) | |
| Fire Season, (11/06 to 3/07) | Males, 65-74 years | 0-4 | 3.03 | (-6.37, 13.38) | | 10.58 | (-6.13, 30.25) | | -0.10 | (-30.10, 42.77) | |
| Fire Season, (11/06 to 3/07) | Males, 65-74 years | 0-8 | 3.31 | (-6.11, 13.67) | | 12.02 | (-5.42, 32.69) | | 13.73 | (-20.13, 61.95) | |
| Fire Season, (11/06 to 3/07) | Males, 65-74 years | 0-12 | 2.80 | (-6.77, 13.35) | | 8.79 | (-7.99, 28.62) | | 19.57 | (-18.16, 74.70) | |
| Fire Season, (11/06 to 3/07) | Males, 65-74 years | 0-24 | 3.04 | (-7.66, 14.98) | | 6.32 | (-11.02, 27.03) | | 15.20 | (-25.48, 78.07) | |
| Fire Season, (11/06 to 3/07) | Males, 65-74 years | 0-48 | 2.94 | (-10.26, 18.08) | | 4.85 | (-15.58, 30.23) | | 17.88 | (-29.40, 96.82) | |
| Fire Season, (11/06 to 3/07) | Males, 75+ years | 0 | 2.38 | (-2.47, 7.47) | | 4.54 | (-3.87, 13.68) | | 6.11 | (-12.53, 28.71) | |
| Fire Season, (11/06 to 3/07) | Males, 75+ years | 1 | 3.55 | (-1.65, 9.03) | | 5.48 | (-3.39, 15.16) | | 10.27 | (-10.11, 35.25) | |
| Fire Season, (11/06 to 3/07) | Males, 75+ years | 2 | 1.75 | (-3.55, 7.35) | | 1.78 | (-6.93, 11.30) | | 15.96 | (-5.73, 42.63) | |
| Fire Season, (11/06 to 3/07) | Males, 75+ years | 0-2 | 2.91 | (-2.17, 8.26) | | 4.76 | (-4.09, 14.43) | | 8.20 | (-11.57, 32.37) | |
| Fire Season, (11/06 to 3/07) | Males, 75+ years | 0-3 | 2.69 | (-2.58, 8.24) | | 4.08 | (-5.01, 14.03) | | 11.50 | (-9.46, 37.33) | |
| Fire Season, (11/06 to 3/07) | Males, 75+ years | 0-4 | 2.98 | (-2.51, 8.78) | | 5.05 | (-4.47, 15.51) | | 12.02 | (-10.13, 39.63) | |
| Fire Season, (11/06 to 3/07) | Males, 75+ years | 0-8 | 3.97 | (-1.73, 10.01) | | 7.45 | (-2.77, 18.74) | | 21.64 | (-4.83, 55.47) | |
| Fire Season, (11/06 to 3/07) | Males, 75+ years | 0-12 | 4.13 | (-1.82, 10.44) | | 8.86 | (-1.83, 20.71) | | 23.01 | (-5.42, 59.98) | |
| Fire Season, (11/06 to 3/07) | Males, 75+ years | 0-24 | 4.65 | (-2.16, 11.93) | | 9.22 | (-2.45, 22.30) | | 22.21 | (-9.01, 64.14) | |
| Fire Season, (11/06 to 3/07) | Males, 75+ years | 0-48 | 9.41 | (1.00, 18.53) | ** | 14.13 | (-0.08, 30.36) | * | 37.68 | (-0.33, 90.17) | * |
| Fire Season, (11/06 to 3/07) | Females, 35-64 years | 0 | 1.00 | (-5.27, 7.67) | | 0.21 | (-10.56, 12.27) | | -8.58 | (-30.98, 21.10) | |
| Fire Season, (11/06 to 3/07) | Females, 35-64 years | 1 | -1.04 | (-7.83, 6.24) | | -3.77 | (-15.38, 9.44) | | -16.59 | (-40.50, 16.92) | |
| Fire Season, (11/06 to 3/07) | Females, 35-64 years | 2 | -1.32 | (-8.67, 6.61) | | -2.25 | (-14.26, 11.44) | | -15.18 | (-41.61, 23.21) | |
| Fire Season, (11/06 to 3/07) | Females, 35-64 years | 0-2 | 0.11 | (-6.46, 7.15) | | -1.47 | (-12.75, 11.27) | | -11.20 | (-34.88, 21.08) | |

| Period[a] | Stratification | Lag (hours) | $PM_{2.5}$ % difference (95% CI) | | $PM_{10}$ % difference (95% CI) | | CO % % difference (95% CI) | |
|---|---|---|---|---|---|---|---|---|
| Fire Season, (11/06 to 3/07) | Females, 35-64 years | 0-3 | -0.42 | (-7.28, 6.96) | -1.95 | (-13.64, 11.32) | -13.87 | (-38.76, 21.13) |
| Fire Season, (11/06 to 3/07) | Females, 35-64 years | 0-4 | -0.68 | (-7.75, 6.94) | -2.22 | (-14.17, 11.39) | -14.64 | (-40.21, 21.88) |
| Fire Season, (11/06 to 3/07) | Females, 35-64 years | 0-8 | 0.28 | (-7.10, 8.23) | -0.91 | (-13.50, 13.51) | -14.83 | (-41.97, 25.00) |
| Fire Season, (11/06 to 3/07) | Females, 35-64 years | 0-12 | 1.97 | (-6.11, 10.74) | 0.91 | (-12.92, 16.92) | -3.42 | (-34.27, 41.91) |
| Fire Season, (11/06 to 3/07) | Females, 35-64 years | 0-24 | 3.96 | (-6.60, 15.71) | 4.74 | (-12.97, 26.05) | 13.25 | (-30.10, 83.51) |
| Fire Season, (11/06 to 3/07) | Females, 35-64 years | 0-48 | 1.90 | (-13.22, 19.65) | -0.82 | (-23.64, 28.83) | 27.25 | (-31.50, 136.40) |
| Fire Season, (11/06 to 3/07) | Females, 65-74 years | 0 | 4.65 | (-6.35, 16.95) | 1.69 | (-16.66, 24.08) | 26.74 | (-14.83, 88.60) |
| Fire Season, (11/06 to 3/07) | Females, 65-74 years | 1 | 3.76 | (-6.70, 15.39) | 6.04 | (-11.90, 27.62) | 3.82 | (-30.33, 54.72) |
| Fire Season, (11/06 to 3/07) | Females, 65-74 years | 2 | 4.10 | (-6.94, 16.46) | 3.17 | (-14.86, 25.02) | -2.08 | (-36.79, 51.69) |
| Fire Season, (11/06 to 3/07) | Females, 65-74 years | 0-2 | 4.47 | (-6.36, 16.55) | 4.32 | (-14.07, 26.65) | 19.64 | (-18.73, 76.14) |
| Fire Season, (11/06 to 3/07) | Females, 65-74 years | 0-3 | 4.49 | (-6.54, 16.84) | 3.95 | (-14.87, 26.93) | 11.25 | (-25.93, 67.09) |
| Fire Season, (11/06 to 3/07) | Females, 65-74 years | 0-4 | 5.14 | (-6.24, 17.90) | 5.03 | (-14.76, 29.42) | 8.72 | (-29.25, 67.07) |
| Fire Season, (11/06 to 3/07) | Females, 65-74 years | 0-8 | 10.27 | (-3.68, 26.23) | 10.45 | (-12.55, 39.50) | 9.17 | (-34.83, 82.86) |
| Fire Season, (11/06 to 3/07) | Females, 65-74 years | 0-12 | 11.32 | (-2.88, 27.61) | 12.47 | (-10.78, 41.78) | 12.34 | (-36.19, 97.78) |
| Fire Season, (11/06 to 3/07) | Females, 65-74 years | 0-24 | 10.99 | (-3.72, 27.94) | 12.91 | (-11.89, 44.70) | 24.95 | (-35.93, 143.68) |
| Fire Season, (11/06 to 3/07) | Females, 65-74 years | 0-48 | 12.20 | (-6.87, 35.18) | 10.39 | (-18.91, 50.28) | 23.71 | (-43.53, 170.99) |
| Fire Season, (11/06 to 3/07) | Females, 75+ years | 0 | -0.23 | (-6.80, 6.81) | 0.08 | (-10.74, 12.20) | -4.96 | (-25.80, 21.75) |
| Fire Season, (11/06 to 3/07) | Females, 75+ years | 1 | -0.71 | (-7.68, 6.80) | 5.92 | (-5.53, 18.76) | -8.06 | (-30.41, 21.47) |
| Fire Season, (11/06 to 3/07) | Females, 75+ years | 2 | -0.18 | (-6.99, 7.12) | 2.22 | (-8.43, 14.11) | -6.63 | (-29.60, 23.84) |
| Fire Season, (11/06 to 3/07) | Females, 75+ years | 0-2 | -0.71 | (-7.64, 6.73) | 3.05 | (-8.39, 15.93) | -7.79 | (-29.76, 21.03) |
| Fire Season, (11/06 to 3/07) | Females, 75+ years | 0-3 | -0.58 | (-7.67, 7.05) | 2.97 | (-8.75, 16.20) | -10.59 | (-33.29, 19.85) |
| Fire Season, (11/06 to 3/07) | Females, 75+ years | 0-4 | -0.23 | (-7.37, 7.46) | 3.99 | (-7.93, 17.45) | -9.51 | (-33.31, 22.78) |
| Fire Season, (11/06 to 3/07) | Females, 75+ years | 0-8 | 0.67 | (-7.45, 9.51) | 8.66 | (-4.96, 24.23) | -12.95 | (-39.16, 24.53) |
| Fire Season, (11/06 to 3/07) | Females, 75+ years | 0-12 | -1.61 | (-9.73, 7.24) | 6.25 | (-6.92, 21.28) | -12.91 | (-38.93, 24.20) |
| Fire Season, (11/06 to 3/07) | Females, 75+ years | 0-24 | -4.41 | (-13.10, 5.16) | 0.67 | (-12.70, 16.09) | -12.14 | (-39.17, 26.90) |
| Fire Season, (11/06 to 3/07) | Females, 75+ years | 0-48 | -3.70 | (-13.07, 6.68) | -0.50 | (-14.87, 16.30) | -7.19 | (-36.18, 34.98) |

| Period[a] | Stratification | Lag (hours) | $PM_{2.5}$ % difference (95% CI) | | | $PM_{10}$ % difference (95% CI) | | | CO % % difference (95% CI) | | |
|---|---|---|---|---|---|---|---|---|---|---|---|
| Fire Season, (11/06 to 3/07) | All data | 0 | 9.13 | (-4.75, 25.03) | | -5.66 | (-17.86, 8.37) | | -5.11 | (-12.02, 2.34) | |
| Fire Season, (11/06 to 3/07) | All data | 1 | 6.72 | (-6.68, 22.03) | | -1.76 | (-14.71, 13.14) | | -3.22 | (-10.19, 4.30) | |
| Fire Season, (11/06 to 3/07) | All data | 2 | 9.73 | (-3.41, 24.65) | | -7.92 | (-20.67, 6.88) | | -4.00 | (-10.87, 3.41) | |
| Fire Season, (11/06 to 3/07) | All data | 0-2 | 8.59 | (-5.31, 24.53) | | -3.11 | (-15.98, 11.73) | | -4.94 | (-12.05, 2.74) | |
| Fire Season, (11/06 to 3/07) | All data | 0-3 | 10.14 | (-4.06, 26.45) | | -5.22 | (-18.48, 10.19) | | -5.75 | (-13.18, 2.33) | |
| Fire Season, (11/06 to 3/07) | All data | 0-4 | 11.45 | (-2.99, 28.05) | | -6.32 | (-19.95, 9.63) | | -5.50 | (-13.25, 2.94) | |
| Fire Season, (11/06 to 3/07) | All data | 0-8 | 19.99 | (3.85, 38.64) | ** | -4.96 | (-20.55, 13.70) | | -6.76 | (-15.38, 2.74) | |
| Fire Season, (11/06 to 3/07) | All data | 0-12 | 24.13 | (5.38, 46.22) | # | -2.39 | (-19.71, 18.66) | | -8.59 | (-17.86, 1.72) | * |
| Fire Season, (11/06 to 3/07) | All data | 0-24 | 15.24 | (-5.23, 40.12) | | 0.61 | (-20.00, 26.52) | | -7.19 | (-18.85, 6.15) | |
| Fire Season, (11/06 to 3/07) | All data | 0-48 | 8.07 | (-12.49, 33.46) | | 16.36 | (-11.89, 53.69) | | -10.65 | (-24.78, 6.15) | |
| Fire Season, (11/06 to 3/07) | 35-64 years | 0 | 2.42 | (-17.64, 27.35) | | -6.97 | (-25.59, 16.31) | | -8.26 | (-20.33, 5.63) | |
| Fire Season, (11/06 to 3/07) | 35-64 years | 1 | -5.47 | (-24.48, 18.31) | | 0.86 | (-19.49, 26.37) | | -2.55 | (-14.07, 10.53) | |
| Fire Season, (11/06 to 3/07) | 35-64 years | 2 | -0.34 | (-19.79, 23.83) | | -17.48 | (-36.80, 7.73) | | 3.84 | (-7.75, 16.89) | |
| Fire Season, (11/06 to 3/07) | 35-64 years | 0-2 | -2.48 | (-22.30, 22.39) | | -0.61 | (-20.71, 24.59) | | -5.52 | (-17.72, 8.49) | |
| Fire Season, (11/06 to 3/07) | 35-64 years | 0-3 | -1.90 | (-22.29, 23.84) | | -5.40 | (-26.06, 21.03) | | -2.44 | (-15.07, 12.06) | |
| Fire Season, (11/06 to 3/07) | 35-64 years | 0-4 | 0.12 | (-20.92, 26.76) | | -11.93 | (-32.61, 15.11) | | -2.75 | (-16.11, 12.74) | |
| Fire Season, (11/06 to 3/07) | 35-64 years | 0-8 | 9.56 | (-14.56, 40.49) | | -19.45 | (-41.57, 11.03) | | -1.45 | (-17.12, 17.18) | |
| Fire Season, (11/06 to 3/07) | 35-64 years | 0-12 | 11.97 | (-15.50, 48.36) | | -13.57 | (-38.83, 22.13) | | -0.15 | (-17.50, 20.85) | |
| Fire Season, (11/06 to 3/07) | 35-64 years | 0-24 | 14.78 | (-18.25, 61.16) | | 8.49 | (-27.22, 61.73) | | 3.84 | (-18.35, 32.06) | |
| Fire Season, (11/06 to 3/07) | 35-64 years | 0-48 | 21.21 | (-15.26, 73.38) | | 33.18 | (-20.37, 122.75) | | -20.40 | (-42.21, 9.65) | |
| Fire Season, (11/06 to 3/07) | 65-74 years | 0 | 48.93 | (2.40, 116.59) | ** | -9.68 | (-34.69, 24.89) | | -9.79 | (-25.56, 9.31) | |
| Fire Season, (11/06 to 3/07) | 65-74 years | 1 | 53.66 | (6.83, 121.00) | ** | -12.94 | (-37.92, 22.08) | | -16.12 | (-31.94, 3.38) | * |
| Fire Season, (11/06 to 3/07) | 65-74 years | 2 | 41.04 | (1.73, 95.55) | ** | -6.74 | (-32.52, 28.89) | | -7.20 | (-23.28, 12.24) | |
| Fire Season, (11/06 to 3/07) | 65-74 years | 0-2 | 57.00 | (8.31, 127.60) | ** | -11.60 | (-36.94, 23.91) | | -15.01 | (-31.25, 5.06) | |
| Fire Season, (11/06 to 3/07) | 65-74 years | 0-3 | 56.02 | (7.99, 125.40) | ** | -11.67 | (-37.69, 25.21) | | -14.61 | (-31.60, 6.60) | |
| Fire Season, (11/06 to 3/07) | 65-74 years | 0-4 | 56.25 | (8.35, 125.31) | ** | -6.83 | (-34.22, 31.97) | | -16.68 | (-34.08, 5.32) | |

| Period[a] | Stratification | Lag (hours) | $PM_{2.5}$ % difference (95% CI) | | | $PM_{10}$ % difference (95% CI) | | CO % % difference (95% CI) | | |
|---|---|---|---|---|---|---|---|---|---|---|
| Fire Season, (11/06 to 3/07) | 65-74 years | 0-8 | 45.50 | (0.67, 110.30) | ** | 8.89 | (-24.41, 56.87) | -18.72 | (-37.41, 5.53) | |
| Fire Season, (11/06 to 3/07) | 65-74 years | 0-12 | 20.95 | (-18.06, 78.54) | | 20.35 | (-18.99, 78.79) | -18.37 | (-38.01, 7.49) | |
| Fire Season, (11/06 to 3/07) | 65-74 years | 0-24 | -8.72 | (-43.23, 46.77) | | 20.07 | (-29.49, 104.47) | -10.64 | (-34.95, 22.76) | |
| Fire Season, (11/06 to 3/07) | 65-74 years | 0-48 | -25.85 | (-56.97, 27.78) | | 4.04 | (-46.12, 100.90) | -14.22 | (-42.02, 26.92) | |
| Fire Season, (11/06 to 3/07) | 75+ years | 0 | 8.54 | (-11.40, 32.96) | | -4.03 | (-22.56, 18.94) | -1.29 | (-10.91, 9.36) | |
| Fire Season, (11/06 to 3/07) | 75+ years | 1 | 8.48 | (-11.04, 32.28) | | -0.53 | (-20.07, 23.78) | 1.04 | (-9.13, 12.34) | |
| Fire Season, (11/06 to 3/07) | 75+ years | 2 | 11.23 | (-7.61, 33.90) | | -1.98 | (-21.52, 22.41) | -8.14 | (-18.09, 3.01) | |
| Fire Season, (11/06 to 3/07) | 75+ years | 0-2 | 9.31 | (-10.76, 33.91) | | -3.00 | (-22.31, 21.11) | -0.96 | (-10.99, 10.19) | |
| Fire Season, (11/06 to 3/07) | 75+ years | 0-3 | 11.51 | (-8.95, 36.56) | | -3.64 | (-23.55, 21.45) | -4.33 | (-14.93, 7.59) | |
| Fire Season, (11/06 to 3/07) | 75+ years | 0-4 | 11.84 | (-8.62, 36.88) | | -2.91 | (-23.64, 23.45) | -2.93 | (-13.92, 9.46) | |
| Fire Season, (11/06 to 3/07) | 75+ years | 0-8 | 23.38 | (0.45, 51.54) | ** | -3.00 | (-26.29, 27.64) | -5.96 | (-17.68, 7.44) | |
| Fire Season, (11/06 to 3/07) | 75+ years | 0-12 | 38.15 | (9.01, 75.08) | # | -7.72 | (-31.64, 24.56) | -10.31 | (-22.66, 4.02) | |
| Fire Season, (11/06 to 3/07) | 75+ years | 0-24 | 30.28 | (-1.44, 72.22) | * | -14.10 | (-38.75, 20.47) | -13.04 | (-28.21, 5.33) | |
| Fire Season, (11/06 to 3/07) | 75+ years | 0-48 | 13.91 | (-15.60, 53.74) | | 9.18 | (-25.89, 60.84) | -4.86 | (-25.42, 21.36) | |
| Fire Season, (11/06 to 3/07) | Male | 0 | 5.44 | (-11.20, 25.20) | | -6.04 | (-21.00, 11.75) | -8.24 | (-16.36, 0.67) | * |
| Fire Season, (11/06 to 3/07) | Male | 1 | 2.65 | (-13.53, 21.85) | | 1.36 | (-14.97, 20.83) | -5.58 | (-13.92, 3.57) | |
| Fire Season, (11/06 to 3/07) | Male | 2 | 5.98 | (-9.81, 24.54) | | -1.98 | (-18.60, 18.03) | -4.33 | (-12.71, 4.86) | |
| Fire Season, (11/06 to 3/07) | Male | 0-2 | 4.67 | (-12.14, 24.70) | | -1.20 | (-17.26, 17.98) | -8.13 | (-16.58, 1.16) | * |
| Fire Season, (11/06 to 3/07) | Male | 0-3 | 6.10 | (-11.04, 26.55) | | -1.15 | (-18.07, 19.27) | -7.86 | (-16.77, 2.01) | |
| Fire Season, (11/06 to 3/07) | Male | 0-4 | 7.90 | (-9.57, 28.74) | | -1.80 | (-19.22, 19.36) | -7.97 | (-17.33, 2.46) | |
| Fire Season, (11/06 to 3/07) | Male | 0-8 | 11.25 | (-7.23, 33.40) | | 6.28 | (-14.68, 32.40) | -8.19 | (-18.85, 3.87) | |
| Fire Season, (11/06 to 3/07) | Male | 0-12 | 12.40 | (-8.54, 38.14) | | 10.59 | (-12.94, 40.47) | -8.28 | (-19.88, 5.00) | |
| Fire Season, (11/06 to 3/07) | Male | 0-24 | 5.92 | (-17.20, 35.48) | | 13.58 | (-14.28, 50.48) | -8.02 | (-22.24, 8.81) | |
| Fire Season, (11/06 to 3/07) | Male | 0-48 | 4.80 | (-19.52, 36.46) | | 29.35 | (-8.95, 83.76) | -10.87 | (-28.27, 10.74) | |
| Fire Season, (11/06 to 3/07) | Female | 0 | 17.80 | (-5.76, 47.26) | | -5.44 | (-25.01, 19.24) | 2.09 | (-10.93, 17.01) | |
| Fire Season, (11/06 to 3/07) | Female | 1 | 15.51 | (-6.94, 43.38) | | -7.77 | (-27.58, 17.46) | 1.35 | (-10.98, 15.38) | |

| Period[a] | Stratification | Lag (hours) | $PM_{2.5}$ % difference (95% CI) | | | $PM_{10}$ % difference (95% CI) | | | CO % % difference (95% CI) | |
|---|---|---|---|---|---|---|---|---|---|---|
| Fire Season, (11/06 to 3/07) | Female | 2 | 17.58 | (-4.69, 45.05) | | -17.59 | (-36.21, 6.46) | | -3.82 | (-15.38, 9.32) |
| Fire Season, (11/06 to 3/07) | Female | 0-2 | 17.34 | (-5.89, 46.31) | | -7.10 | (-27.07, 18.34) | | 1.65 | (-11.24, 16.41) |
| Fire Season, (11/06 to 3/07) | Female | 0-3 | 19.04 | (-4.78, 48.84) | | -12.49 | (-32.32, 13.15) | | -1.91 | (-14.87, 13.01) |
| Fire Season, (11/06 to 3/07) | Female | 0-4 | 19.41 | (-4.73, 49.67) | | -14.68 | (-34.90, 11.82) | | -1.47 | (-14.64, 13.73) |
| Fire Season, (11/06 to 3/07) | Female | 0-8 | 38.78 | (8.98, 76.73) | # | -23.79 | (-44.52, 4.69) | * | -5.40 | (-19.29, 10.89) |
| Fire Season, (11/06 to 3/07) | Female | 0-12 | 48.98 | (13.40, 95.72) | # | -23.62 | (-45.82, 7.68) | | -10.04 | (-24.53, 7.22) |
| Fire Season, (11/06 to 3/07) | Female | 0-24 | 33.70 | (-3.19, 84.64) | * | -21.62 | (-47.39, 16.77) | | -6.94 | (-25.63, 16.45) |
| Fire Season, (11/06 to 3/07) | Female | 0-48 | 14.13 | (-19.77, 62.34) | | -6.89 | (-41.27, 47.61) | | -12.78 | (-34.40, 15.96) |
| Fire Season, (11/06 to 3/07) | Males, 35-64 years | 0 | -8.58 | (-30.27, 19.85) | | -6.40 | (-28.66, 22.79) | | -8.18 | (-21.91, 7.97) |
| Fire Season, (11/06 to 3/07) | Males, 35-64 years | 1 | -15.19 | (-36.20, 12.72) | | 4.08 | (-20.80, 36.78) | | -3.32 | (-16.75, 12.28) |
| Fire Season, (11/06 to 3/07) | Males, 35-64 years | 2 | -6.80 | (-28.45, 21.39) | | -12.05 | (-35.53, 19.97) | | 5.23 | (-8.72, 21.32) |
| Fire Season, (11/06 to 3/07) | Males, 35-64 years | 0-2 | -13.46 | (-35.09, 15.39) | | 1.08 | (-23.22, 33.05) | | -6.48 | (-20.68, 10.26) |
| Fire Season, (11/06 to 3/07) | Males, 35-64 years | 0-3 | -12.49 | (-34.80, 17.45) | | -2.17 | (-27.36, 31.76) | | -2.33 | (-17.31, 15.35) |
| Fire Season, (11/06 to 3/07) | Males, 35-64 years | 0-4 | -10.15 | (-33.12, 20.70) | | -7.73 | (-32.86, 26.79) | | -2.04 | (-17.92, 16.91) |
| Fire Season, (11/06 to 3/07) | Males, 35-64 years | 0-8 | -4.32 | (-29.84, 30.48) | | -6.74 | (-36.05, 36.01) | | 3.70 | (-15.97, 27.99) |
| Fire Season, (11/06 to 3/07) | Males, 35-64 years | 0-12 | -4.94 | (-32.97, 34.82) | | 0.95 | (-32.68, 51.39) | | 7.67 | (-14.28, 35.24) |
| Fire Season, (11/06 to 3/07) | Males, 35-64 years | 0-24 | 5.91 | (-29.85, 59.88) | | 25.03 | (-20.88, 97.59) | | 8.55 | (-18.18, 44.02) |
| Fire Season, (11/06 to 3/07) | Males, 35-64 years | 0-48 | 19.90 | (-21.84, 83.92) | | 44.74 | (-20.76, 164.38) | | -21.57 | (-46.23, 14.41) |
| Fire Season, (11/06 to 3/07) | Males, 65-74 years | 0 | 23.61 | (-23.72, 100.32) | | -22.58 | (-50.22, 20.40) | | -10.48 | (-28.23, 11.66) |
| Fire Season, (11/06 to 3/07) | Males, 65-74 years | 1 | 23.15 | (-22.37, 95.35) | | -21.00 | (-49.12, 22.67) | | -16.73 | (-34.34, 5.61) |
| Fire Season, (11/06 to 3/07) | Males, 65-74 years | 2 | 24.20 | (-18.25, 88.67) | | -6.56 | (-39.24, 43.71) | | -4.93 | (-22.69, 16.91) |
| Fire Season, (11/06 to 3/07) | Males, 65-74 years | 0-2 | 30.34 | (-18.84, 109.32) | | -23.78 | (-51.52, 19.84) | | -16.64 | (-34.85, 6.66) |
| Fire Season, (11/06 to 3/07) | Males, 65-74 years | 0-3 | 31.04 | (-18.32, 110.23) | | -20.45 | (-50.34, 27.43) | | -13.89 | (-32.92, 10.53) |
| Fire Season, (11/06 to 3/07) | Males, 65-74 years | 0-4 | 34.03 | (-16.02, 113.89) | | -11.82 | (-44.78, 40.81) | | -15.83 | (-35.48, 9.80) |
| Fire Season, (11/06 to 3/07) | Males, 65-74 years | 0-8 | 21.74 | (-22.75, 91.84) | | 17.24 | (-25.64, 84.86) | | -15.60 | (-37.04, 13.14) |
| Fire Season, (11/06 to 3/07) | Males, 65-74 years | 0-12 | 0.89 | (-37.75, 63.51) | | 39.23 | (-13.57, 124.30) | | -11.92 | (-35.52, 20.33) |

| Period[a] | Stratification | Lag (hours) | $PM_{2.5}$ % difference (95% CI) | | | $PM_{10}$ % difference (95% CI) | | | CO % % difference (95% CI) | | |
|---|---|---|---|---|---|---|---|---|---|---|---|
| Fire Season, (11/06 to 3/07) | Males, 65-74 years | 0-24 | -26.53 | (-59.31, 32.65) | | 51.53 | (-19.38, 184.83) | | -10.10 | (-37.88, 30.11) | |
| Fire Season, (11/06 to 3/07) | Males, 65-74 years | 0-48 | -33.40 | (-66.04, 30.62) | | 30.44 | (-40.14, 184.22) | | -10.95 | (-43.55, 40.47) | |
| Fire Season, (11/06 to 3/07) | Males, 75+ years | 0 | 18.79 | (-8.60, 54.39) | | 0.10 | (-23.04, 30.21) | | -6.29 | (-17.89, 6.96) | |
| Fire Season, (11/06 to 3/07) | Males, 75+ years | 1 | 17.42 | (-9.15, 51.75) | | 8.21 | (-17.36, 41.68) | | -1.56 | (-14.27, 13.02) | |
| Fire Season, (11/06 to 3/07) | Males, 75+ years | 2 | 14.58 | (-9.92, 45.74) | | 9.35 | (-17.47, 44.89) | | -11.25 | (-24.14, 3.82) | |
| Fire Season, (11/06 to 3/07) | Males, 75+ years | 0-2 | 19.01 | (-8.56, 54.89) | | 5.49 | (-19.50, 38.22) | | -4.91 | (-17.11, 9.08) | |
| Fire Season, (11/06 to 3/07) | Males, 75+ years | 0-3 | 20.01 | (-7.60, 55.88) | | 7.20 | (-19.19, 42.21) | | -7.93 | (-21.04, 7.36) | |
| Fire Season, (11/06 to 3/07) | Males, 75+ years | 0-4 | 20.04 | (-7.48, 55.76) | | 7.50 | (-19.98, 44.40) | | -7.31 | (-21.13, 8.93) | |
| Fire Season, (11/06 to 3/07) | Males, 75+ years | 0-8 | 23.26 | (-5.08, 60.07) | | 10.14 | (-21.74, 55.02) | | -11.22 | (-26.08, 6.64) | |
| Fire Season, (11/06 to 3/07) | Males, 75+ years | 0-12 | 36.25 | (0.69, 84.37) | ** | 1.23 | (-31.27, 49.09) | | -16.45 | (-31.99, 2.64) | * |
| Fire Season, (11/06 to 3/07) | Males, 75+ years | 0-24 | 28.28 | (-10.74, 84.35) | | -12.78 | (-44.34, 36.67) | | -19.35 | (-37.92, 4.77) | |
| Fire Season, (11/06 to 3/07) | Males, 75+ years | 0-48 | 12.87 | (-23.64, 66.82) | | 18.85 | (-29.63, 100.75) | | -2.17 | (-29.81, 36.34) | |
| Fire Season, (11/06 to 3/07) | Females, 35-64 years | 0 | 34.13 | (-9.12, 97.95) | | -7.50 | (-37.67, 37.28) | | -8.49 | (-31.35, 21.98) | |
| Fire Season, (11/06 to 3/07) | Females, 35-64 years | 1 | 19.76 | (-17.25, 73.33) | | -5.96 | (-37.56, 41.65) | | -1.23 | (-22.08, 25.21) | |
| Fire Season, (11/06 to 3/07) | Females, 35-64 years | 2 | 19.62 | (-19.00, 76.65) | | -28.76 | (-58.56, 22.47) | | 0.89 | (-18.96, 25.60) | |
| Fire Season, (11/06 to 3/07) | Females, 35-64 years | 0-2 | 26.52 | (-13.70, 85.48) | | -3.61 | (-35.50, 44.04) | | -3.29 | (-25.22, 25.07) | |
| Fire Season, (11/06 to 3/07) | Females, 35-64 years | 0-3 | 26.37 | (-14.80, 87.43) | | -11.06 | (-43.18, 39.22) | | -2.75 | (-24.53, 25.33) | |
| Fire Season, (11/06 to 3/07) | Females, 35-64 years | 0-4 | 28.75 | (-14.28, 93.39) | | -20.35 | (-52.26, 32.91) | | -4.64 | (-27.47, 25.37) | |
| Fire Season, (11/06 to 3/07) | Females, 35-64 years | 0-8 | 49.45 | (-3.37, 131.14) | * | -43.04 | (-70.00, 8.15) | * | -12.16 | (-35.74, 20.06) | |
| Fire Season, (11/06 to 3/07) | Females, 35-64 years | 0-12 | 62.20 | (-1.23, 166.38) | * | -41.41 | (-70.70, 17.16) | | -18.33 | (-42.97, 16.97) | |
| Fire Season, (11/06 to 3/07) | Females, 35-64 years | 0-24 | 41.85 | (-22.89, 160.94) | | -31.53 | (-70.09, 56.75) | | -11.26 | (-44.16, 41.01) | |
| Fire Season, (11/06 to 3/07) | Females, 35-64 years | 0-48 | 31.66 | (-32.58, 157.10) | | 1.44 | (-62.40, 173.66) | | -21.51 | (-57.39, 44.59) | |
| Fire Season, (11/06 to 3/07) | Females, 65-74 years | 0 | 97.64 | (3.97, 275.70) | ** | 10.33 | (-30.75, 75.77) | | -5.32 | (-36.57, 41.34) | |
| Fire Season, (11/06 to 3/07) | Females, 65-74 years | 1 | 131.99 | (14.80, 368.78) | ** | 2.73 | (-37.00, 67.51) | | -12.70 | (-44.47, 37.25) | |
| Fire Season, (11/06 to 3/07) | Females, 65-74 years | 2 | 77.22 | (-1.49, 218.84) | * | -6.29 | (-42.57, 52.90) | | -14.64 | (-45.20, 32.94) | |
| Fire Season, (11/06 to 3/07) | Females, 65-74 years | 0-2 | 115.15 | (9.91, 321.17) | ** | 8.09 | (-32.68, 73.54) | | -7.53 | (-40.22, 43.05) | |

| Period[a] | Stratification | Lag (hours) | $PM_{2.5}$ % difference (95% CI) | | | $PM_{10}$ % difference (95% CI) | | CO % % difference (95% CI) | |
|---|---|---|---|---|---|---|---|---|---|
| Fire Season, (11/06 to 3/07) | Females, 65-74 years | 0-3 | 109.94 | (7.45, 310.18) | ** | 1.31 | (-37.89, 65.24) | -15.21 | (-47.64, 37.32) |
| Fire Season, (11/06 to 3/07) | Females, 65-74 years | 0-4 | 104.34 | (5.71, 295.00) | ** | 0.59 | (-39.31, 66.73) | -17.40 | (-49.72, 35.68) |
| Fire Season, (11/06 to 3/07) | Females, 65-74 years | 0-8 | 105.37 | (6.22, 297.05) | ** | -2.94 | (-48.09, 81.48) | -25.54 | (-57.74, 31.20) |
| Fire Season, (11/06 to 3/07) | Females, 65-74 years | 0-12 | 70.79 | (-11.61, 229.99) | | -12.13 | (-58.54, 86.26) | -33.47 | (-62.98, 19.55) |
| Fire Season, (11/06 to 3/07) | Females, 65-74 years | 0-24 | 40.85 | (-35.47, 207.43) | | -33.92 | (-76.44, 85.29) | -10.00 | (-52.07, 68.98) |
| Fire Season, (11/06 to 3/07) | Females, 65-74 years | 0-48 | 0.49 | (-60.53, 155.82) | | -39.91 | (-82.69, 108.55) | -19.84 | (-63.09, 74.11) |
| Fire Season, (11/06 to 3/07) | Females, 75+ years | 0 | -4.70 | (-31.30, 32.21) | | -11.60 | (-38.65, 27.38) | 8.63 | (-8.56, 29.05) |
| Fire Season, (11/06 to 3/07) | Females, 75+ years | 1 | -3.52 | (-29.71, 32.43) | | -14.33 | (-40.82, 24.01) | 5.13 | (-10.99, 24.17) |
| Fire Season, (11/06 to 3/07) | Females, 75+ years | 2 | 6.18 | (-20.75, 42.27) | | -16.91 | (-42.03, 19.09) | -4.20 | (-19.01, 13.31) |
| Fire Season, (11/06 to 3/07) | Females, 75+ years | 0-2 | -2.82 | (-29.57, 34.08) | | -17.33 | (-43.48, 20.92) | 6.07 | (-10.91, 26.29) |
| Fire Season, (11/06 to 3/07) | Females, 75+ years | 0-3 | 0.28 | (-27.40, 38.52) | | -20.53 | (-46.29, 17.59) | 1.31 | (-15.74, 21.80) |
| Fire Season, (11/06 to 3/07) | Females, 75+ years | 0-4 | 0.72 | (-27.01, 38.99) | | -18.76 | (-45.69, 21.52) | 3.07 | (-13.93, 23.43) |
| Fire Season, (11/06 to 3/07) | Females, 75+ years | 0-8 | 23.47 | (-11.63, 72.51) | | -21.86 | (-50.43, 23.19) | 0.58 | (-17.23, 22.22) |
| Fire Season, (11/06 to 3/07) | Females, 75+ years | 0-12 | 40.89 | (-3.89, 106.53) | * | -19.39 | (-49.85, 29.55) | -2.91 | (-21.69, 20.37) |
| Fire Season, (11/06 to 3/07) | Females, 75+ years | 0-24 | 32.00 | (-14.95, 104.88) | | -16.07 | (-49.88, 40.53) | -5.33 | (-28.72, 25.74) |
| Fire Season, (11/06 to 3/07) | Females, 75+ years | 0-48 | 14.35 | (-28.57, 83.07) | | -2.36 | (-45.28, 74.22) | -8.13 | (-35.83, 31.51) |

IQRs: $PM_{2.5}$ = 6.1 μg/m$^3$; $PM_{10}$ = 13.7 μg/m$^3$; CO = 0.3 ppm

[a]All data: July 2006–June 2007; Fire season: November 2006–March 2007

*$p < 0.1$; **$p < 0.05$; #$p < 0.01$.
